# Supplementary material for: PIF/harbinger transposon-derived protein promotes 7SL expression to enhance pathogen resistance
Source: EMBO Rep. 2025 Jan 30;26(5):1196–211. doi: 10.1038/s44319-025-00379-8 (PMC11893794; doi:10.1038/s44319-025-00379-8)
Supplement: Supplementary file 1 — Table EV1 [file 44319_2025_379_MOESM1_ESM.docx]

**Table EV1.** PCR primer information in this study.

| **Primer** | **Sequences (5’-3’)** |
| --- | --- |
|  | **Primers for Real-time PCR** |
| 7SL-RT-F | AGGCTGGCGGACCGTTTGAG |
| 7SL-RT-R | AGACTGTCCCGCTGTATT |
| ADF-1L-RT-F | TCGTGGGAAGGGTGGTGTA |
| ADF-1L-RT-R | CGGGTGGATAATCTCCTGTGA |
| IFN-1-RT-F | TACGATGGCTAATAACTCC |
| IFN-1-RT-R | CATTGACAAAGTGCTCCA |
| TNF-α-RT-F | GTTTGCTTGGTACTGGAATGG |
| TNF-α-RT-R | TGTGGGATGATGATCTGGTTG |
| SCRV-M-RT-F | TCAACCTGGCAAACAACA |
| SCRV-M-RT-R | CCTCGGACCTCTGCTTCT |
| SCRV-G-RT-F | TCTGCCATAAGACTACCTG |
| SCRV-G-RT-R | TCTTGACGGTGATGAATG |
| miR-2187-3p-RT-1F | CGCAGTTACAGGCTATGCT |
| miR-2187-3p-RT-1R | GGTCCAGTTTTTTTTTTTTTTTACAGA |
| miR-133-3p-RT-1F | TGGTCCCCTTCAACCAG |
| miR-133-3p-RT-1R | GGTCCAGTTTTTTTTTTTTTTTACAG |
| miR-29a-3p-RT-1F | CAGTAGCACCATTTGAAATCG |
| miR-29a-3p-RT-1R | TCCAGTTTTTTTTTTTTTTTAACCGA |
| TRAF6-RT-1F | ATGATGGAAAAGGAACGGGAAT |
| TRAF6-RT-1R | TCGGACAGCGAACAGTTAGTGA |
| β-actin-RT-F | GAGCCGCACGCTTCTTT |
| β-actin-RT-R | CTGCTGTAGCCGAGGAC |
| GAPDH-RT-F | TTCACTCCTCCATCTTTGAT |
| GAPDH-RT-R | GTAGCAGGACAATGTGGGT |
|  | **Primers for CHIP** |
| 7SL-23-ChiP1-F | TCCATCGACAGCCTTCA |
| 7SL-23-ChiP1-R | CCCGGTAGCTTGGATTA |
| 7SL-23-ChiP2-F | CTAGAAGCCGCATGGTGA |
| 7SL-23-ChiP2-R | ACCCCTCCTTAGACGACC |
| 7SL-16-F | TTCCTCTGATCTGTAGAG |
| 7SL-16-R | AAACGTGACGTCATCGCG |
| 7SL-17-F | GTTATCGATAGATCGGTAG |
| 7SL-17-R | AGCACCCGATCGACATAGTC |
| 7SL-18-F | GCAGCTGGAGGTGACGATGAC |
| 7SL-18-R | CGATCGACATAGTCCGCTGCA |
| ADF-1L-ChiP-F | GCTGGCTACAAAACAAGT |
| ADF-1L-ChiP-R | CATGGATAGTCTATGAAAGG |
|  | **Primers for probes** |
| 7SL-motif1-F | CCCTGCAGCTGCAGTTTCAT |
| 7SL-motif1-R | ATGAAACTGCAGCTGCAGGG |
| 7SL-motif2-F | CTGAGCTGCAGTGGACTATG |
| 7SL-motif2-R | CATAGTCCACTGCAGCTCAG |
|  | **Primers for plasmid construction** |
| ADF-1L-HindIII-1F | CCCAAGCTTAATTTCAGCATGGACTCTT |
| ADF-1L-EcoRI-1R | CCGGAATTCGTTGACACGCAAGTTTTAT |
| ADF-1L-6xHis-1F | CCAAGAGAATCTCATCATCATCATCATCATTGAATAATAATAAAACTTGC |
| ADF-1L-6xHis-1R | TATTATTATTCAATGATGATGATGATGATGAGATTCTCTTGGGAAGTCCT |
| ADF-1L△MADF-1F | TTCGAGTGTCATCGAGAGGCGGACAGTAACTTC |
| ADF-1L△MADF-1R | CTCTCGATGACACTCGAAAGAGTCCATGCTGAA |
| TFIIIC63-CE-BamHI-1F | CTTGGTACCGAGCTCGGATCCATGGCGGATTCCACGGAC |
| TFIIIC63-CE-Xhol-IR | CCCTCTAGATGCATGCTCGAGTTAAATATAATCCAATATTTCTGTCTCCAT |
| TFIIIC90-BamHI-1F | CTTGGTACCGAGCTCGGATCCATGGCGGCCGCCAGTCCG |
| TFIIIC90-Xhol-IR | CCCTCTAGATGCATGCTCGAGCTAGATCATTGGCGAGTCGCA |
| EZH2-BamHI-1F | CTTGGTACCGAGCTCGGATCCATGGTGCTGACAGGGAAGCG |
| EZH2-Xhol-1R | CCCTCTAGATGCATGCTCGAGTCAGGCGATCTCCATCTCTCG |
| SUZ12a-BamHI-1F | CTTGGTACCGAGCTCGGATCCATGCCATCGGCCAGGAAC |
| SUZ12a-Xhol-1R | CCCTCTAGATGCATGCTCGAGTCAGTCCGTTAGTGCGCTTTT |
| KAT2A-BamHI-1F | CTTGGTACCGAGCTCGGATCCATGTCGGACCCGGCGGCG |
| KAT2A-Xhol-1R | CCCTCTAGATGCATGCTCGAGTTACCCTTTGTCCTTGCTACTGG |
| KAT2B-BamHI-1F | CTTGGTACCGAGCTCGGATCCATGGCCGACAGCGCTGGG |
| KAT2B-Xhol-1R | CCCTCTAGATGCATGCTCGAGCTACTTCTCGATGAGGCCTGCT |
| KAT2B-△HAT-1F | AGGATCCTGTCTAAGGACATTAAGGTTCCCAAGG |
| KAT2B-△HAT-1R | GTCCTTAGACAGGATCCTCTTATTGGGCTTCTG |
| TRAF6-1F | GACGATGACGACAAGAAGCTTATGGCTTGCATTGACAGCAAT |
| TRAF6-1R | TGATGGATATCTGCAGAATTCGCACTCCTTAATGTTGAGCTGATTA |
| Ago2-Flag-F | CCCAAGCTTGACAAAATGTATTCCTCTGC |
| Ago2-Flag-R | CGCGGATCCTTTCATCAGTGGGGTCTC |
| 7SL-pcDNA3.1-F | CCCAAGCTTTCGCCGGGTGCGGTGGCGC |
| 7SL-pcDNA3.1-R | CCGGAATTCAAAGACTGGGTCCCGCTGTATTACT |
| 7SL-pmirGLO-F | CCGCTCGAGCGCGTGCCTGTAATCCAA |
| 7SL-pmirGLO-R | GCTCTAGAATCCTCCAGCCTCAGCCTCC |
| 7SL-pmirGLO-mut-F | ACATGAGGATCACCCATGTCTGCAGTGCTCCTGGGGGAGCCCG |
| 7SL-pmirGLO-mut-R | CATGGGTGATCCTCATGTTTTCTAGCCATATCGATACCGAACTTAGTGC |
| 7SL-MS2-F | CCCAAGCTTTCGCCGGGTGCGGTGGCGC |
| 7SL-MS2-R | CCGGAATTCAAAGACTGGGTCCCGCTGTATTACT |
| 7SL-MS2-mut-F | ACATGAGGATCACCCATGTCTGCAGTGCTCCTGGGGGAGCCCG |
| 7SL-MS2-mut-R | CATGGGTGATCCTCATGTTTTCTAGCCATATCGATACCGAACTTAGTGC |
| TRAF6-3’UTR-F | TCTAGTTGTTTAAACGAGCTCTCATCATCATGCTGAGATGTCAGTAC |
| TRAF6-3’UTR-R | TGCCTGCAGGTCGACTCTAGAGCACTCCTTAATGTTGAGCTGATTA |
| TRAF6-3’UTR-mut-F | CAGCAAGTATTTACAGGTTTATGTCGATGTCAATGGGACTT |
| TRAF6-3’UTR-mut-R | CCTGTAAATACTTGCTGTGTCCAGACTTAAAATG |
|  | **Primers for T7 Transcription** |
| 7SL-T7-F | TAATACGACTCACTATAGGGTCGCCGGGTGCGGTGGCG |
| 7SL-T7-R | AAAGACTGGGTCCCGCTGTAT |
| NARL-T7-F | TAATACGACTCACTATAGGGAGCTGCCCGCGCACAACT |
| NARL-T7-R | CTATGCAAACTTTTACGCATGACTC |
