# Supplementary figures and images for: PIF/harbinger transposon-derived protein promotes 7SL expression to enhance pathogen resistance
Source: EMBO Rep. 2025 Jan 30;26(5):1196–211. doi: 10.1038/s44319-025-00379-8 (PMC11893794; doi:10.1038/s44319-025-00379-8)

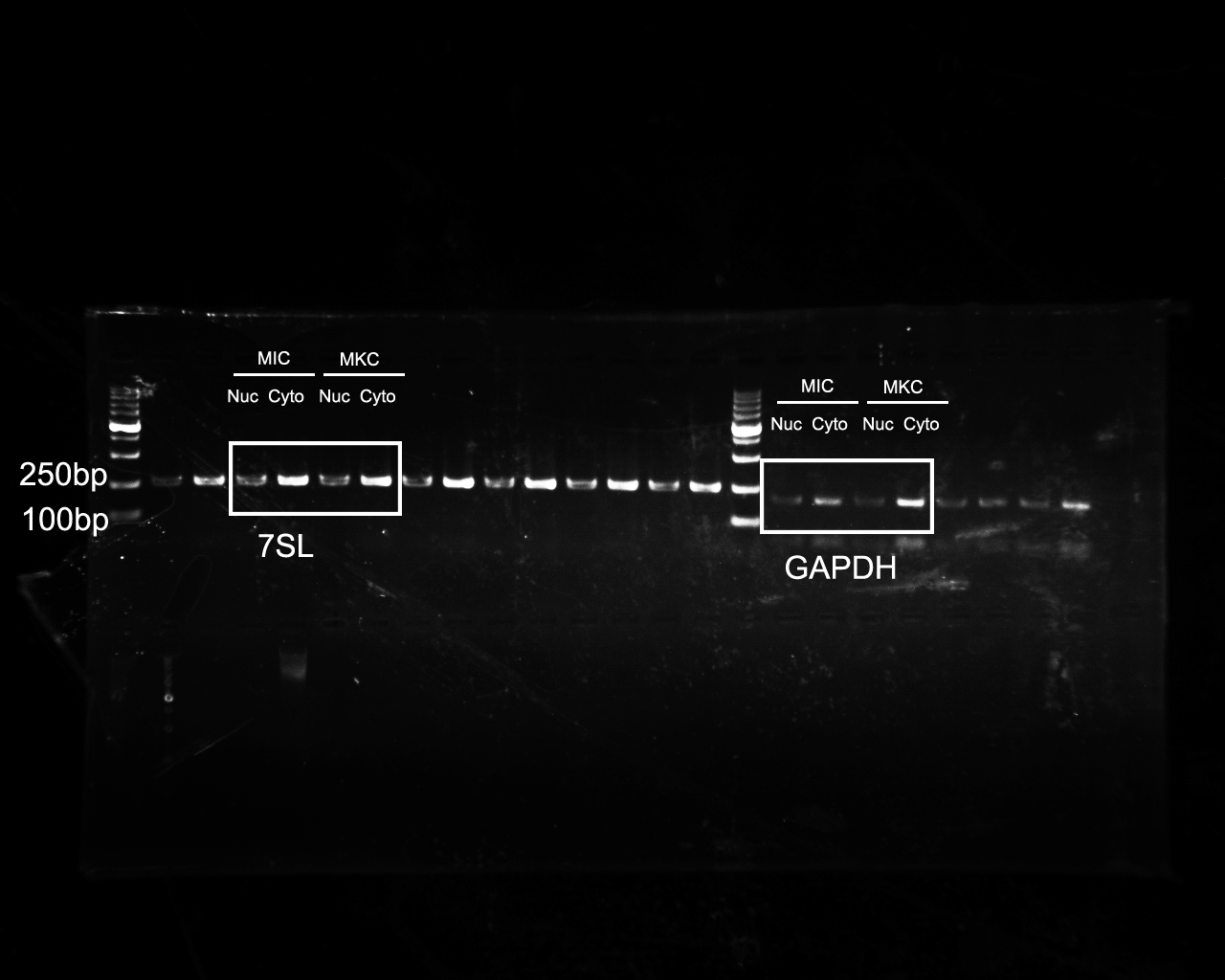

Supplement: Supplementary file 3 — Source data Fig. 1 [file 44319_2025_379_MOESM3_ESM.zip › Figure 1-source data/1G/1G.tif]

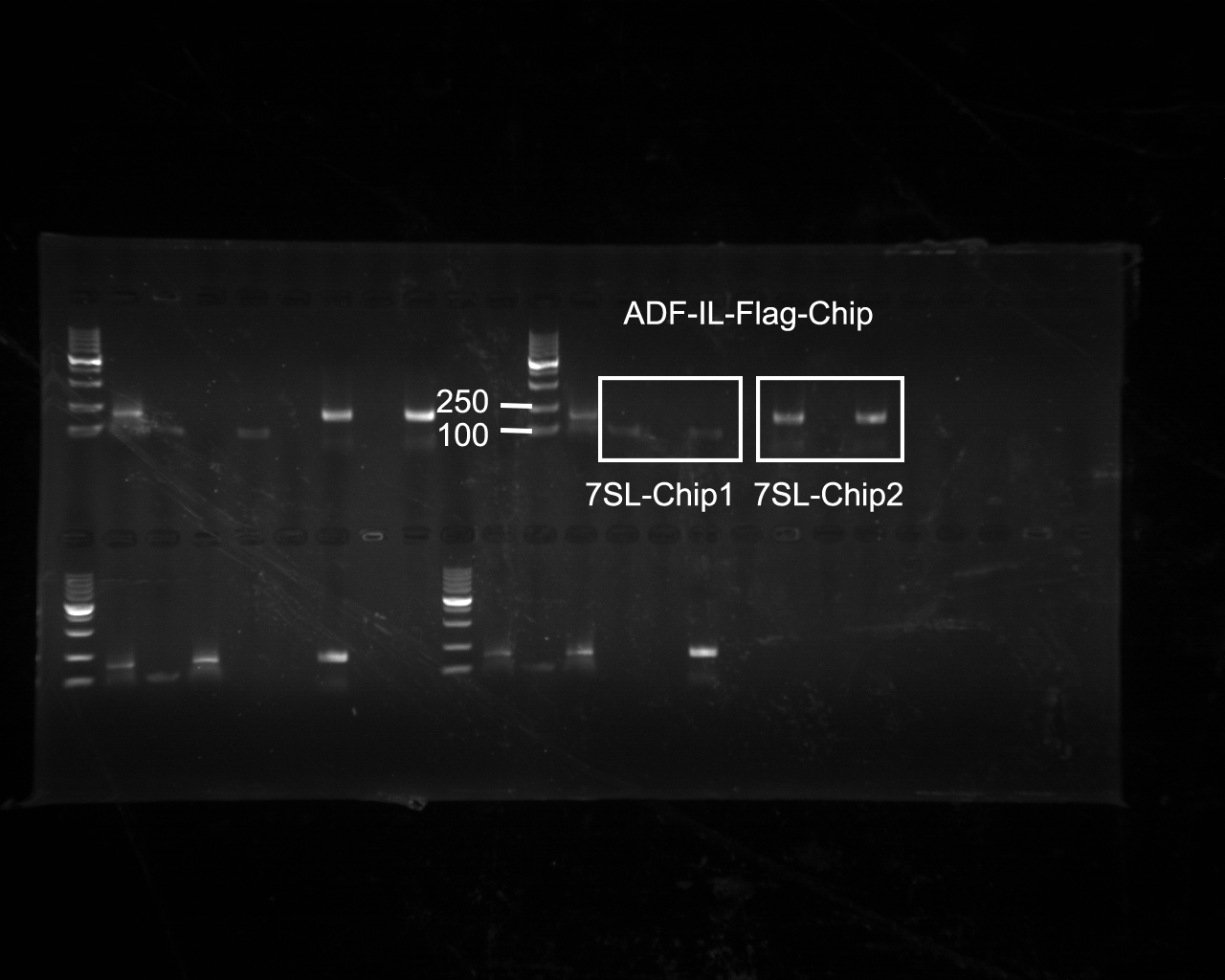

Supplement: Supplementary file 4 — Source data Fig. 2 [file 44319_2025_379_MOESM4_ESM.zip › Figure 2/2B/2B-7SL.tif]

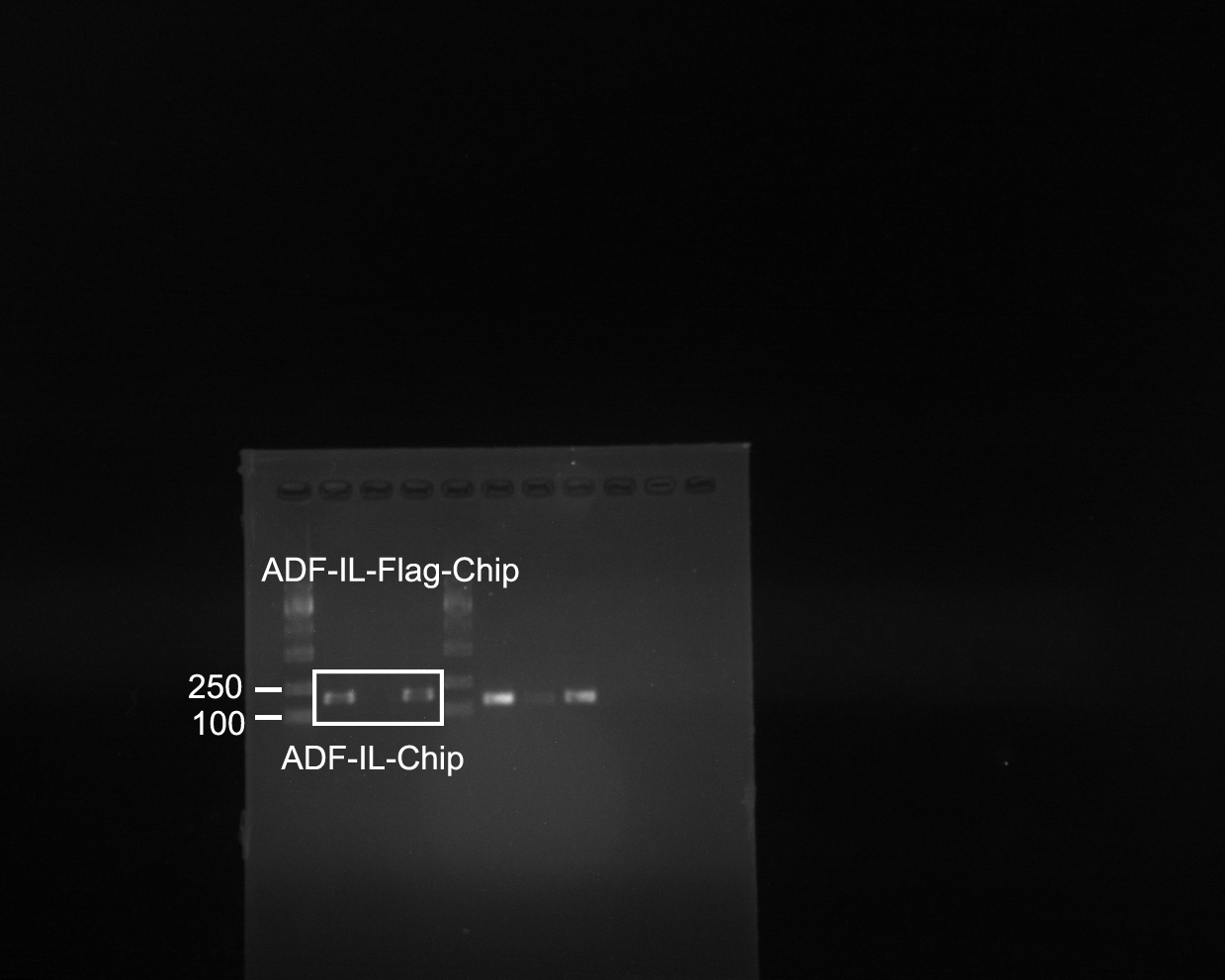

Supplement: Supplementary file 4 — Source data Fig. 2 [file 44319_2025_379_MOESM4_ESM.zip › Figure 2/2B/2B-ADF-IL.tif]

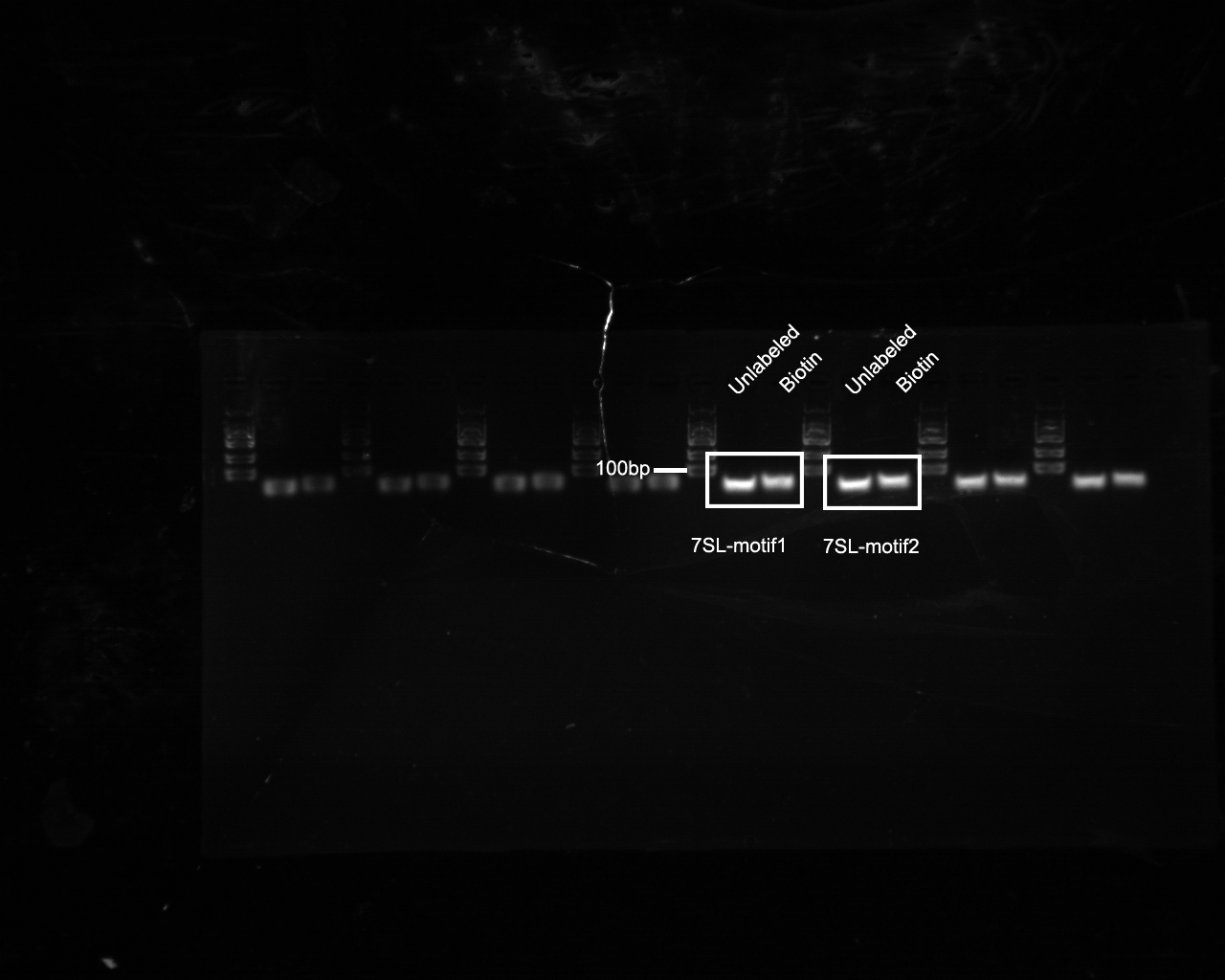

Supplement: Supplementary file 4 — Source data Fig. 2 [file 44319_2025_379_MOESM4_ESM.zip › Figure 2/2C/2C.tif]

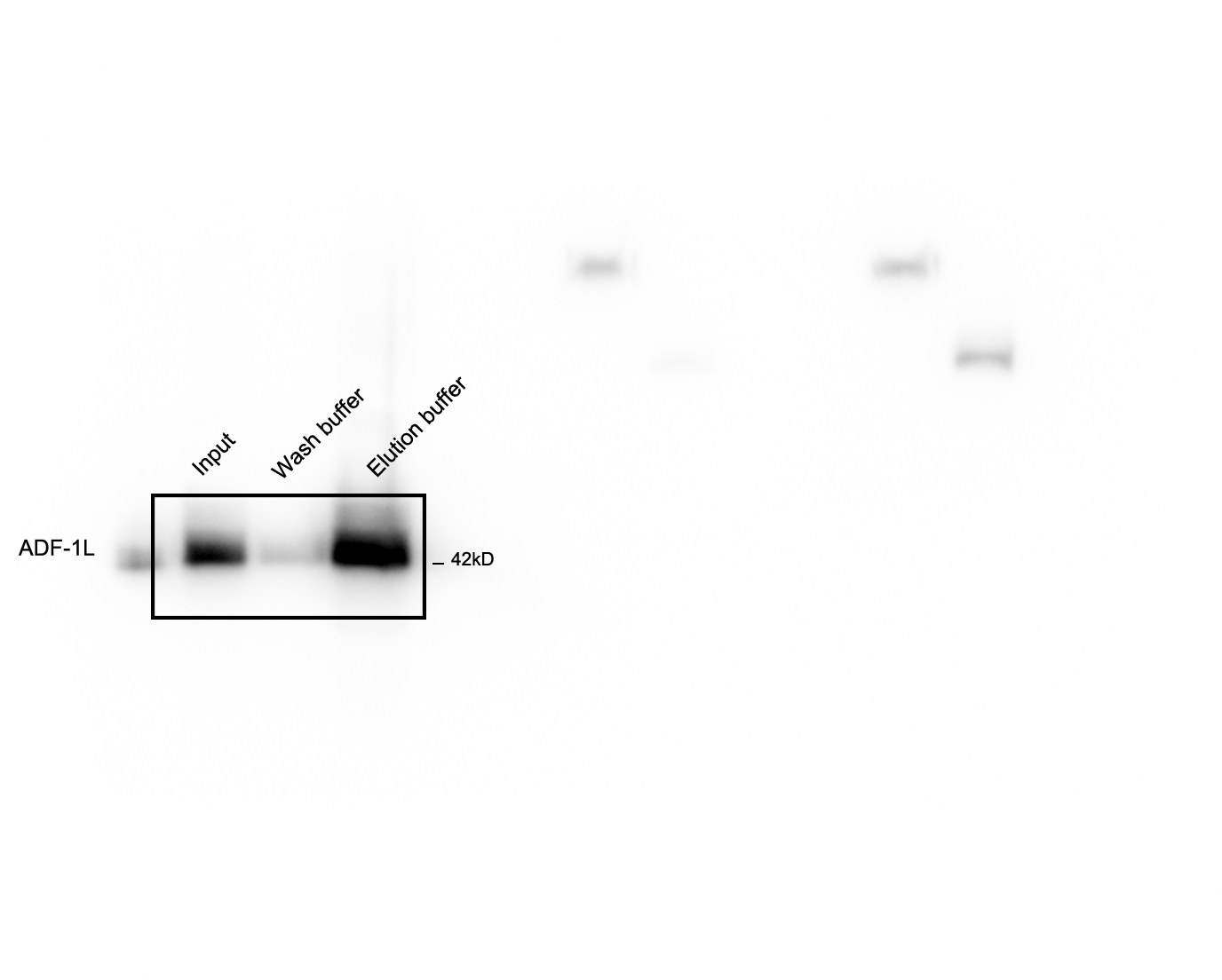

Supplement: Supplementary file 4 — Source data Fig. 2 [file 44319_2025_379_MOESM4_ESM.zip › Figure 2/2D/2D.tif]

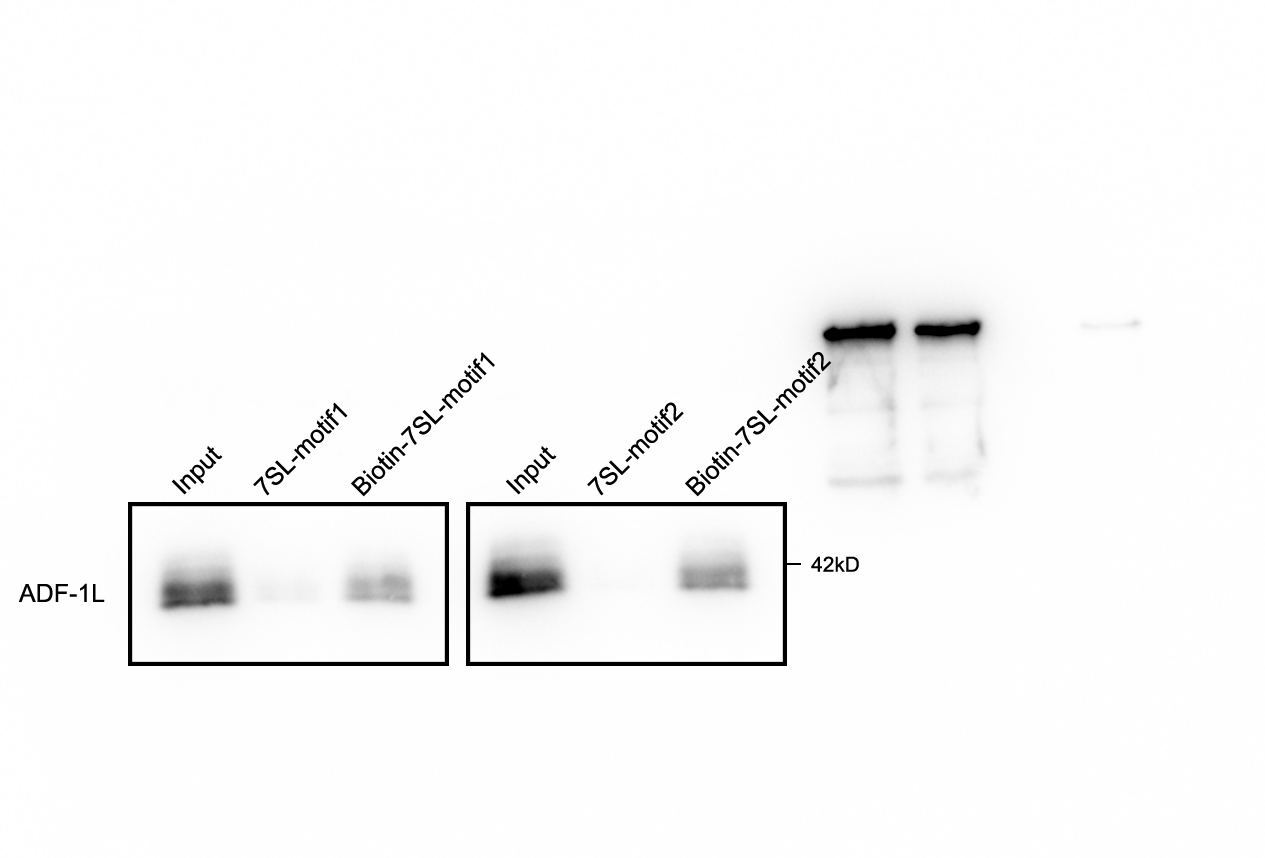

Supplement: Supplementary file 4 — Source data Fig. 2 [file 44319_2025_379_MOESM4_ESM.zip › Figure 2/2E/2E.tif]

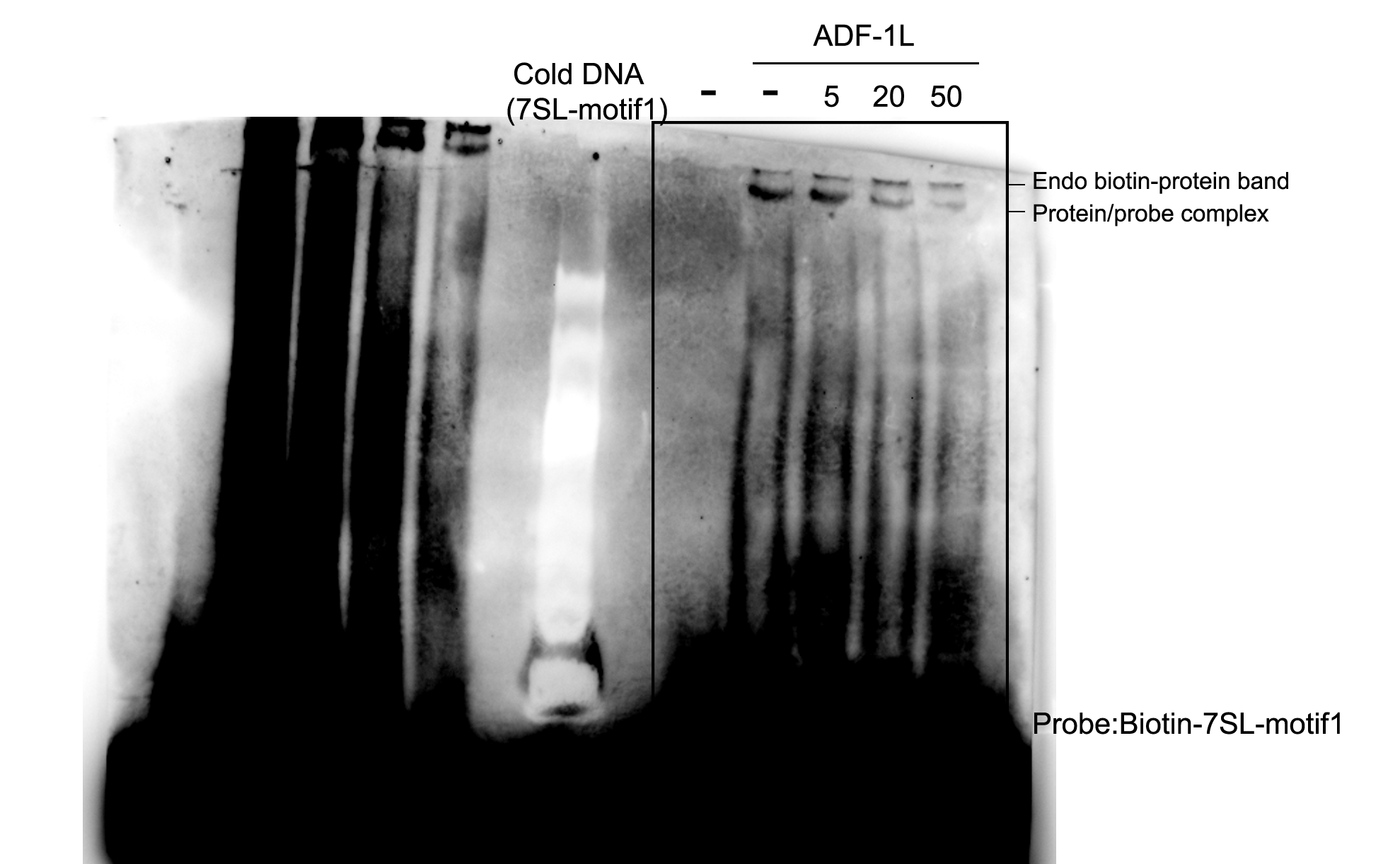

Supplement: Supplementary file 4 — Source data Fig. 2 [file 44319_2025_379_MOESM4_ESM.zip › Figure 2/2F/2F-7SL-motif.tif]

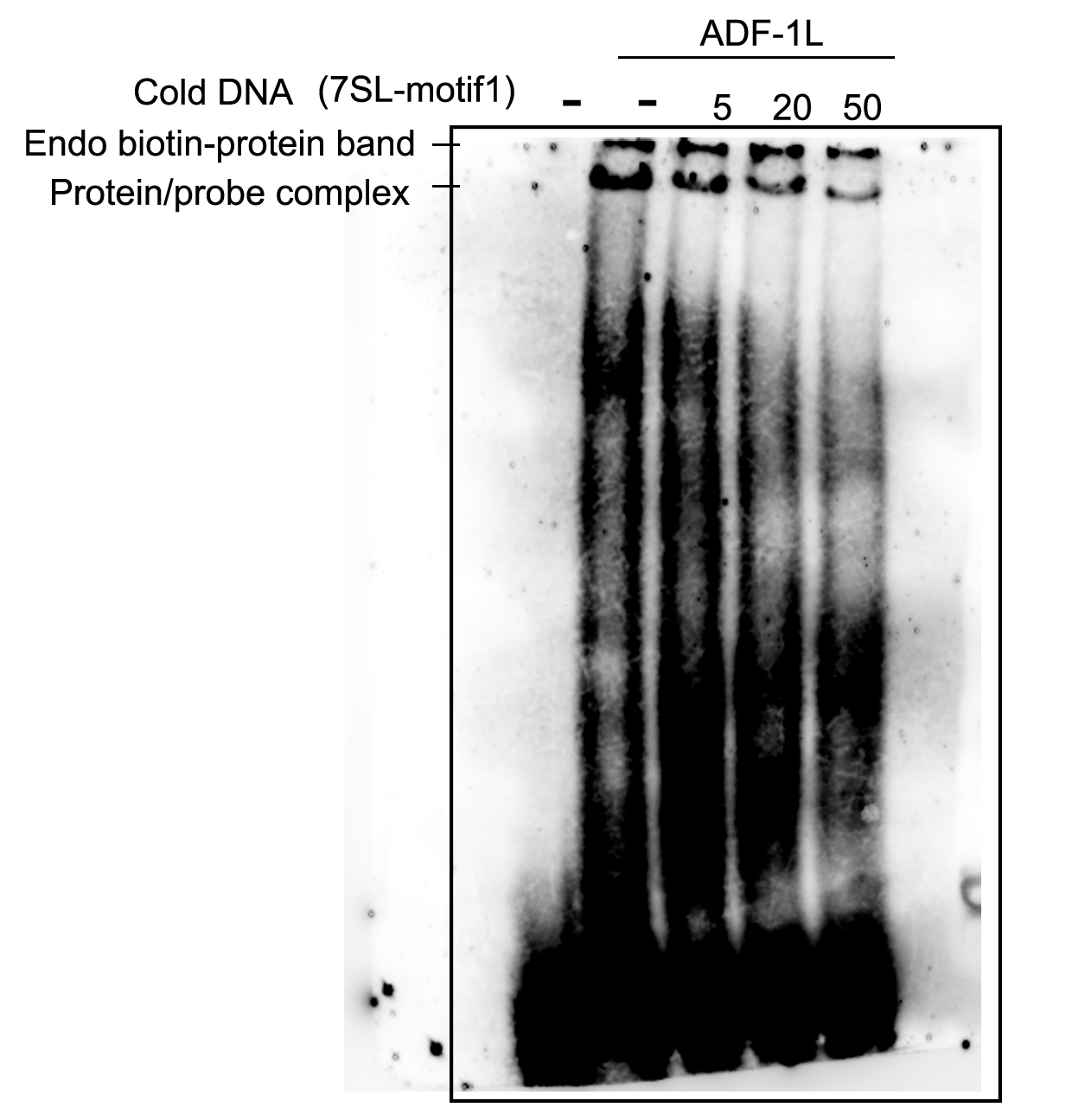

Supplement: Supplementary file 4 — Source data Fig. 2 [file 44319_2025_379_MOESM4_ESM.zip › Figure 2/2F/2F-7SL-motif1.tif]

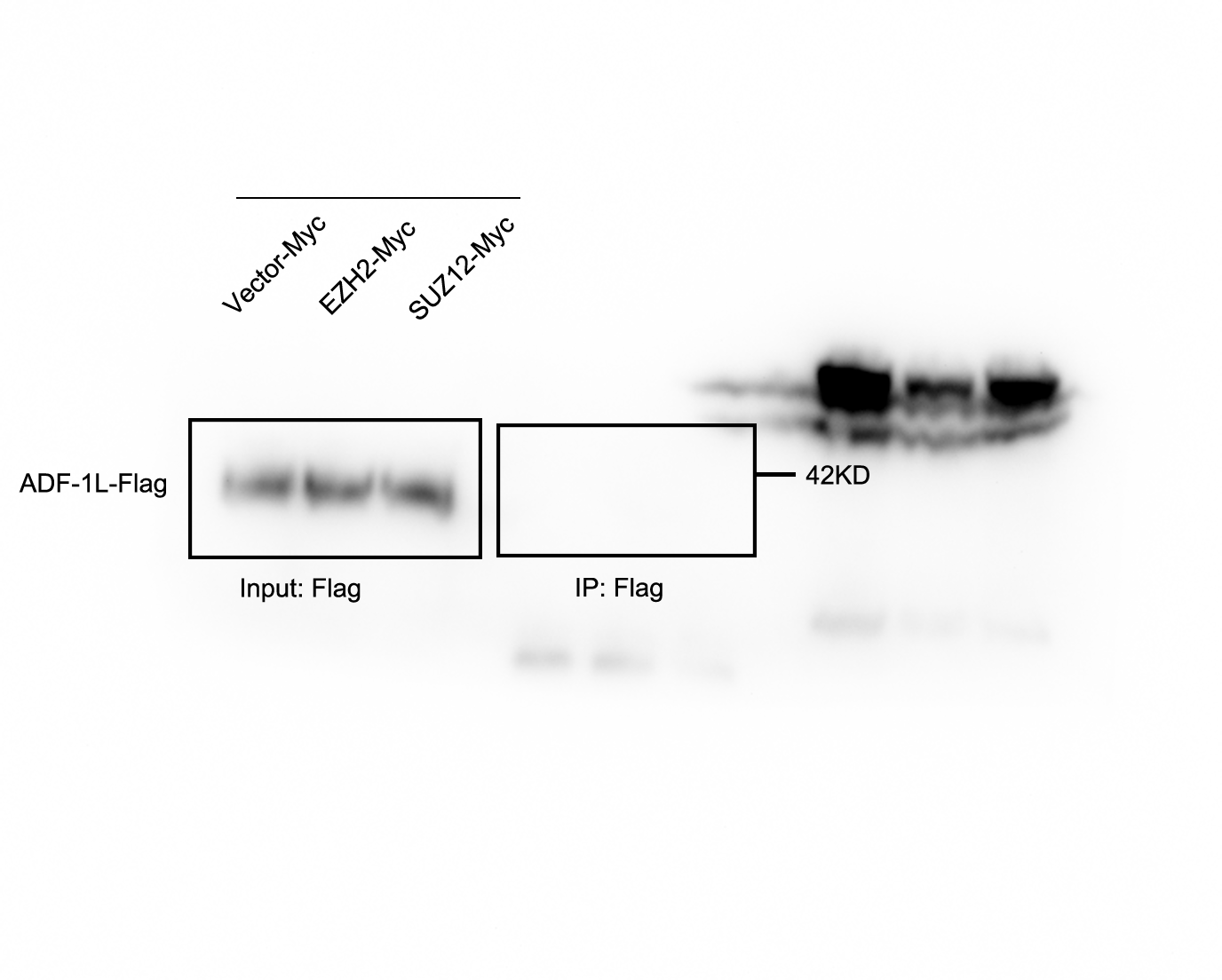

Supplement: Supplementary file 5 — Source data Fig. 3 [file 44319_2025_379_MOESM5_ESM.zip › Figure 3/3A/1A-Flag.tif]

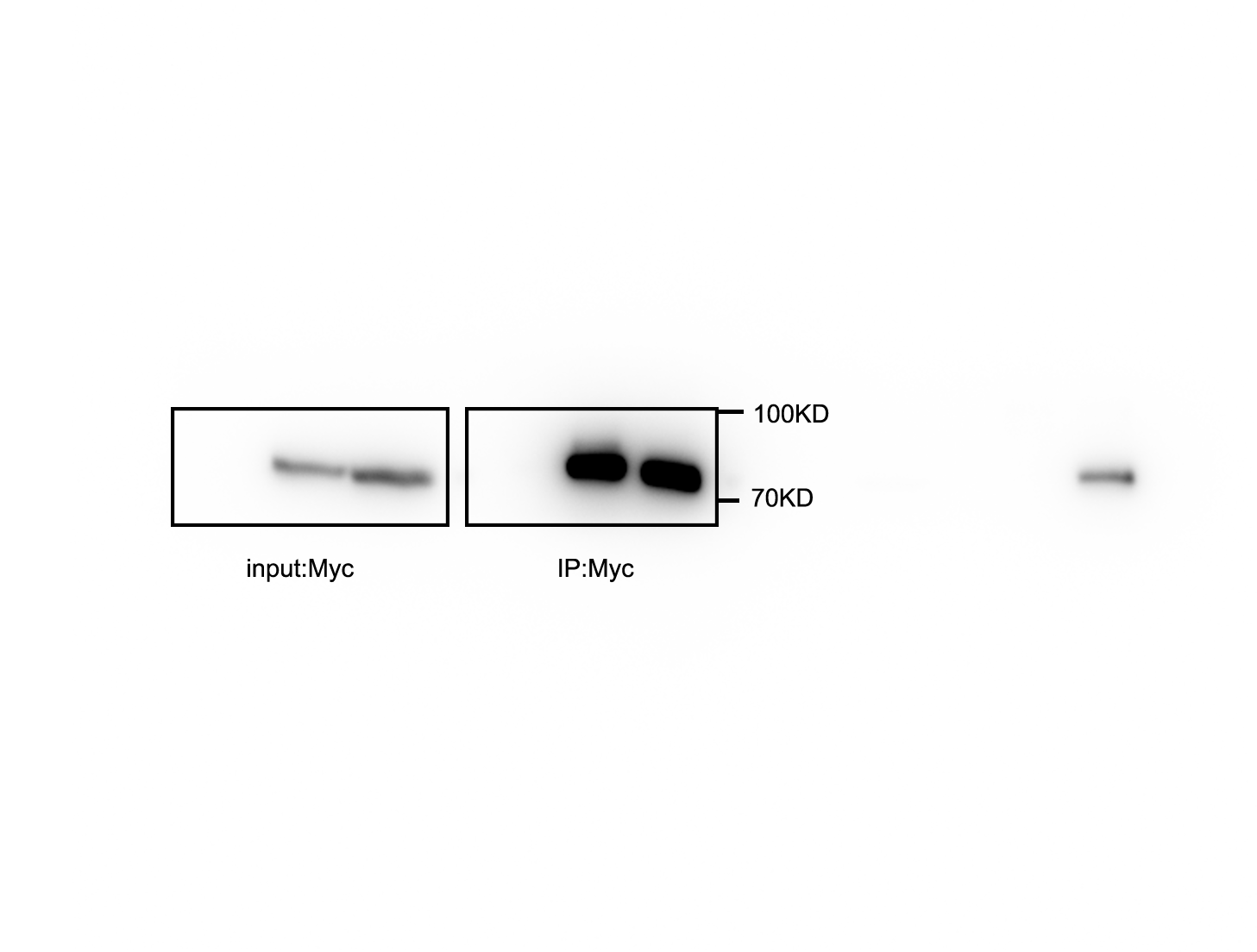

Supplement: Supplementary file 5 — Source data Fig. 3 [file 44319_2025_379_MOESM5_ESM.zip › Figure 3/3A/1A-Myc.tif]

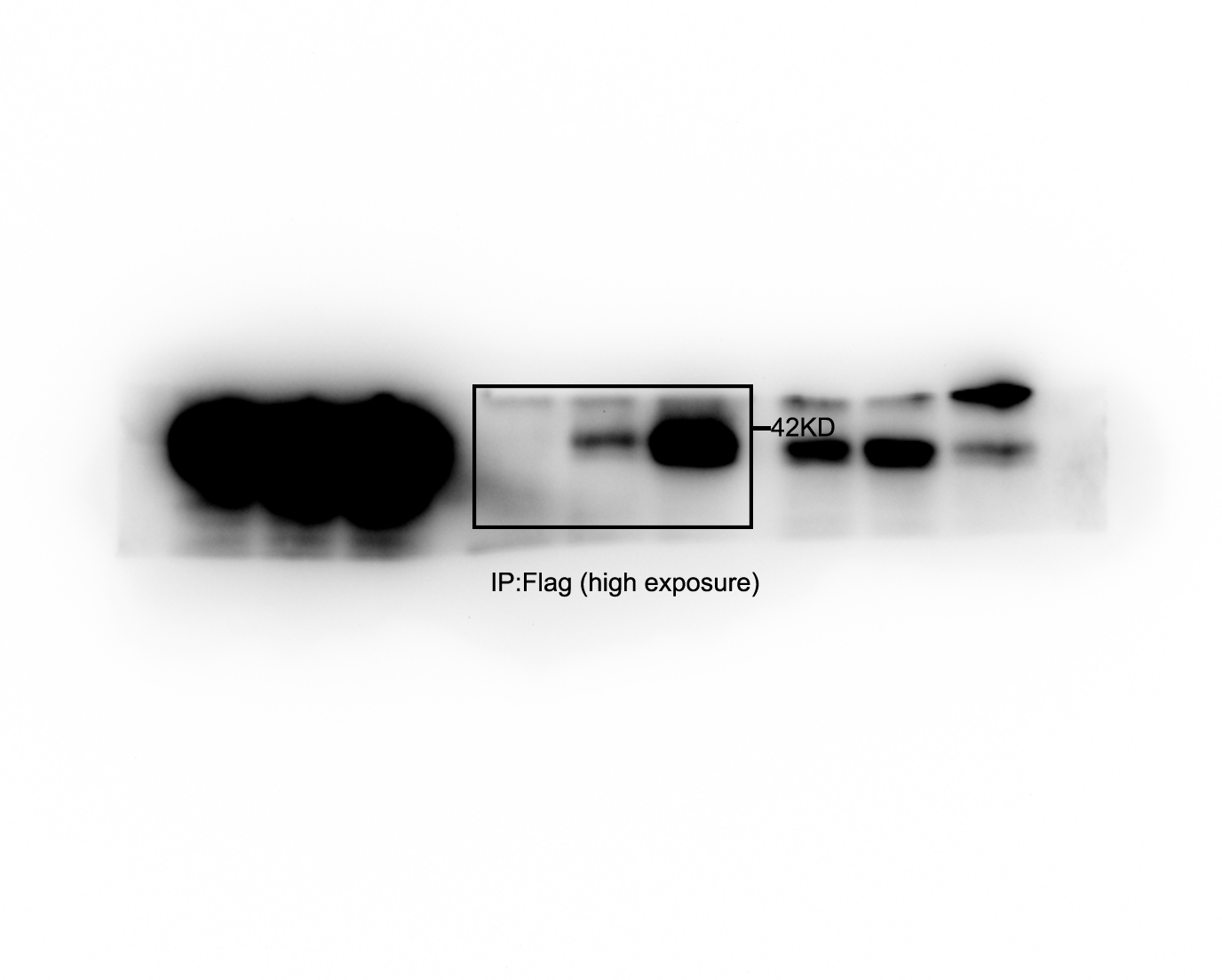

Supplement: Supplementary file 5 — Source data Fig. 3 [file 44319_2025_379_MOESM5_ESM.zip › Figure 3/3B/3B-Flag-high exposure.tif]

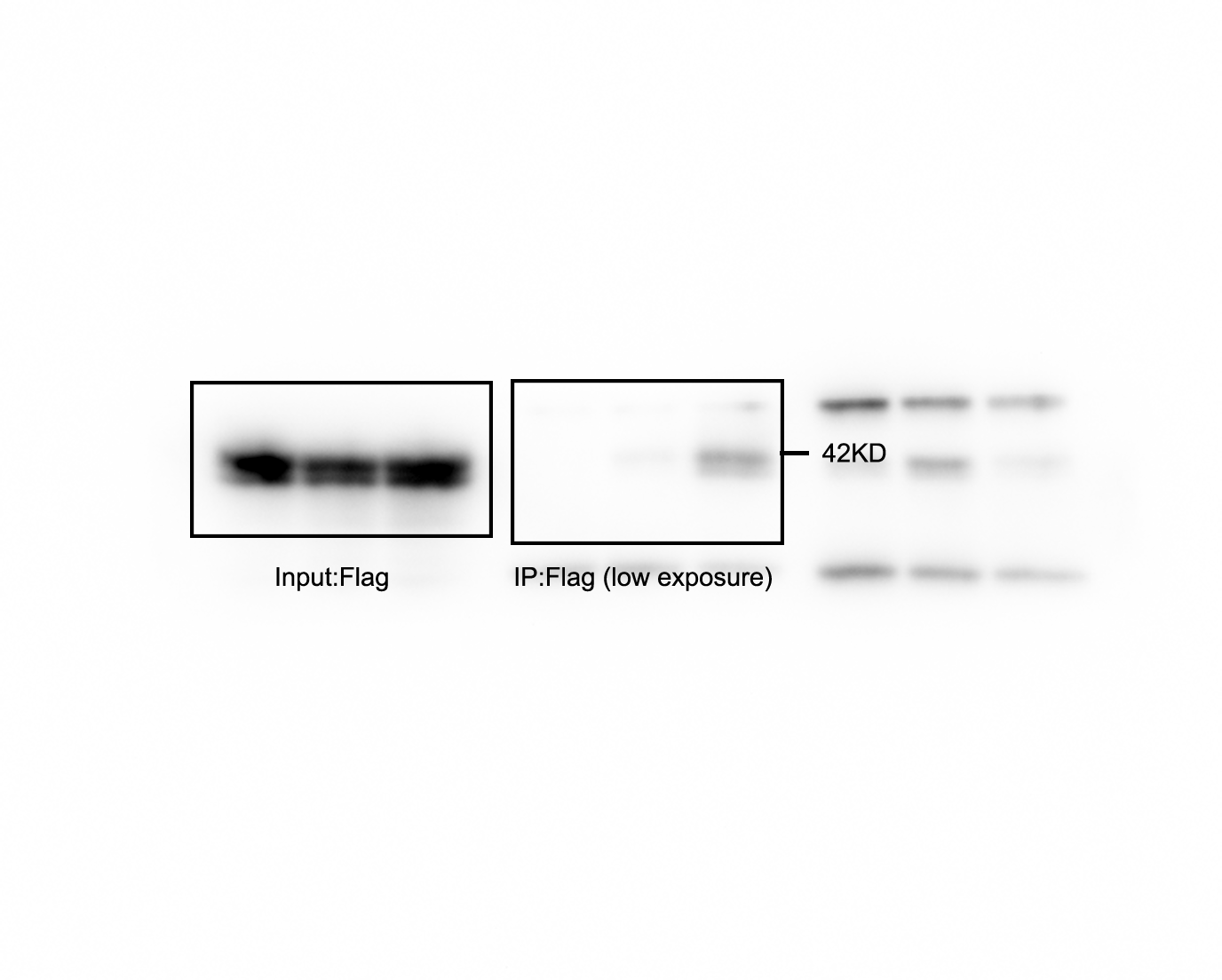

Supplement: Supplementary file 5 — Source data Fig. 3 [file 44319_2025_379_MOESM5_ESM.zip › Figure 3/3B/3B-Flag-low exposure.tif]

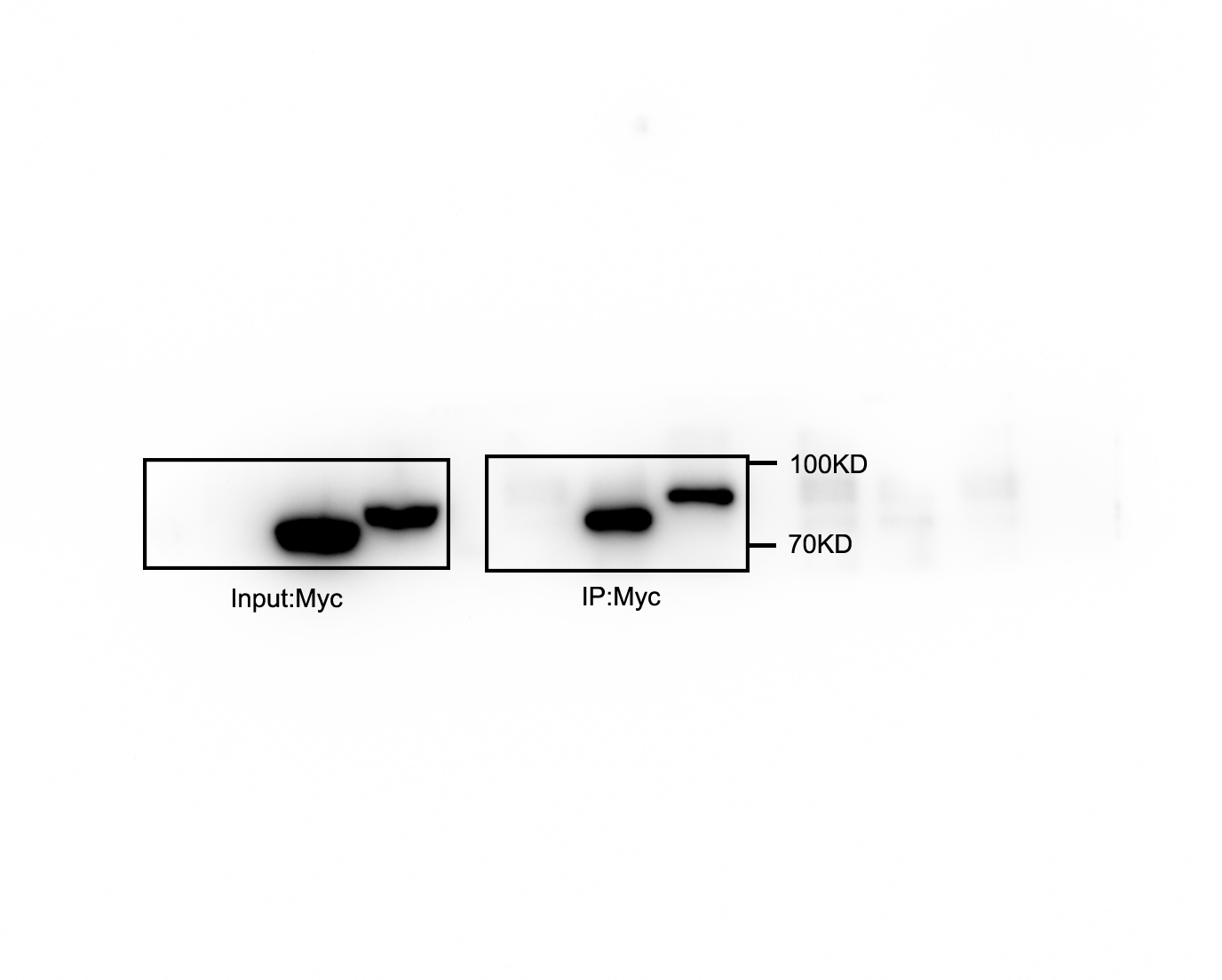

Supplement: Supplementary file 5 — Source data Fig. 3 [file 44319_2025_379_MOESM5_ESM.zip › Figure 3/3B/3B-Myc.tif]

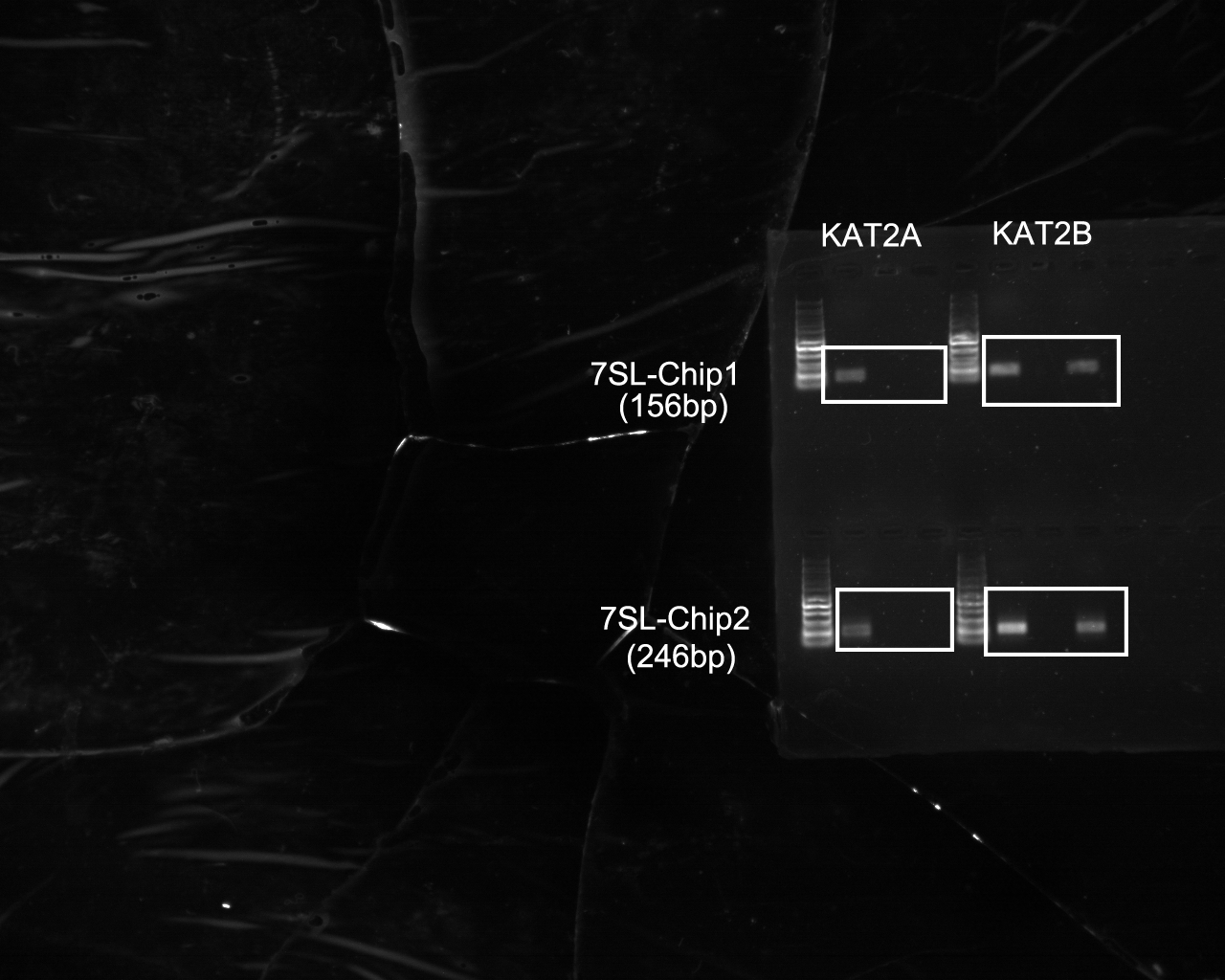

Supplement: Supplementary file 5 — Source data Fig. 3 [file 44319_2025_379_MOESM5_ESM.zip › Figure 3/3C/3C-7SL.tif]

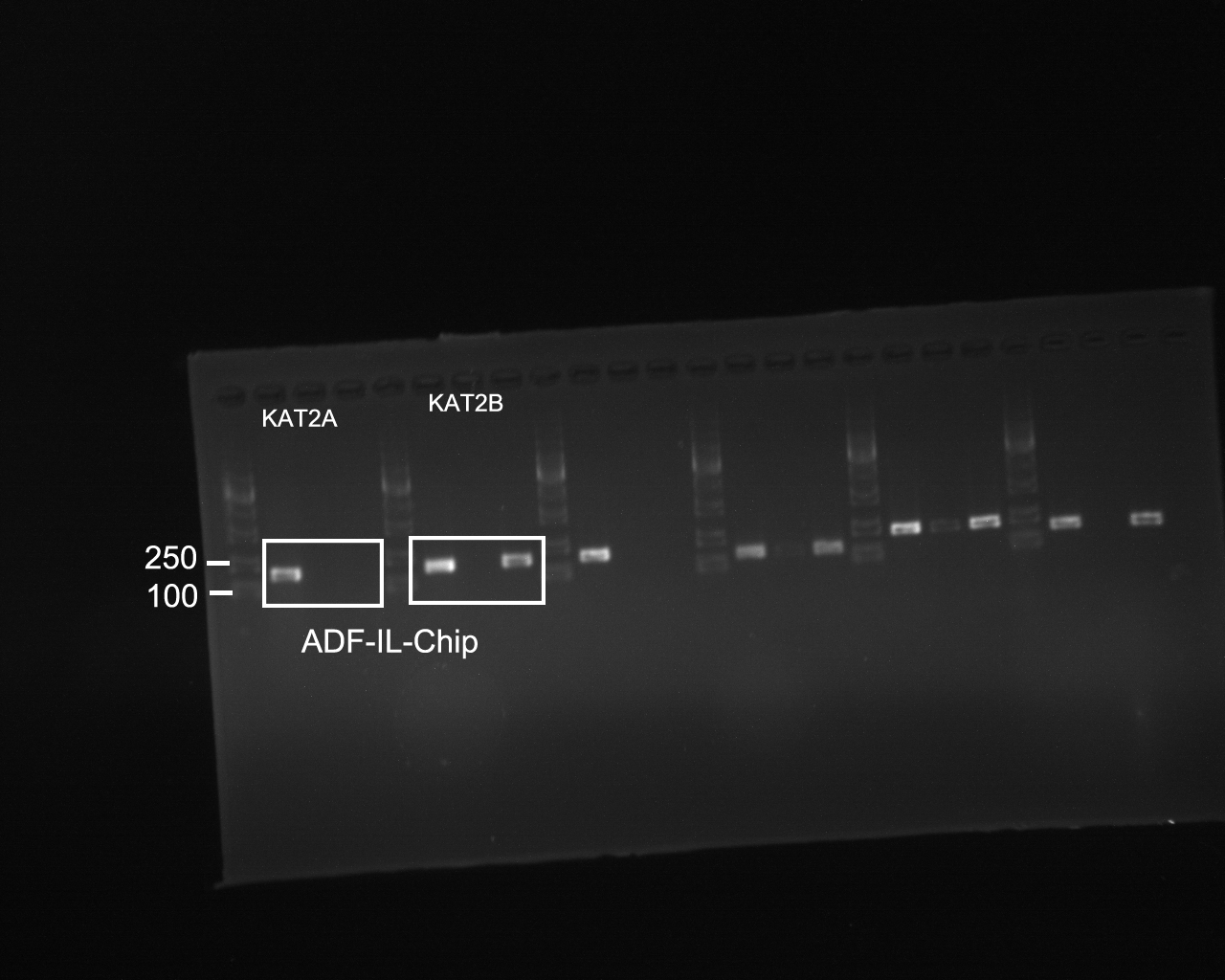

Supplement: Supplementary file 5 — Source data Fig. 3 [file 44319_2025_379_MOESM5_ESM.zip › Figure 3/3C/3C-ADF-IL.tif]

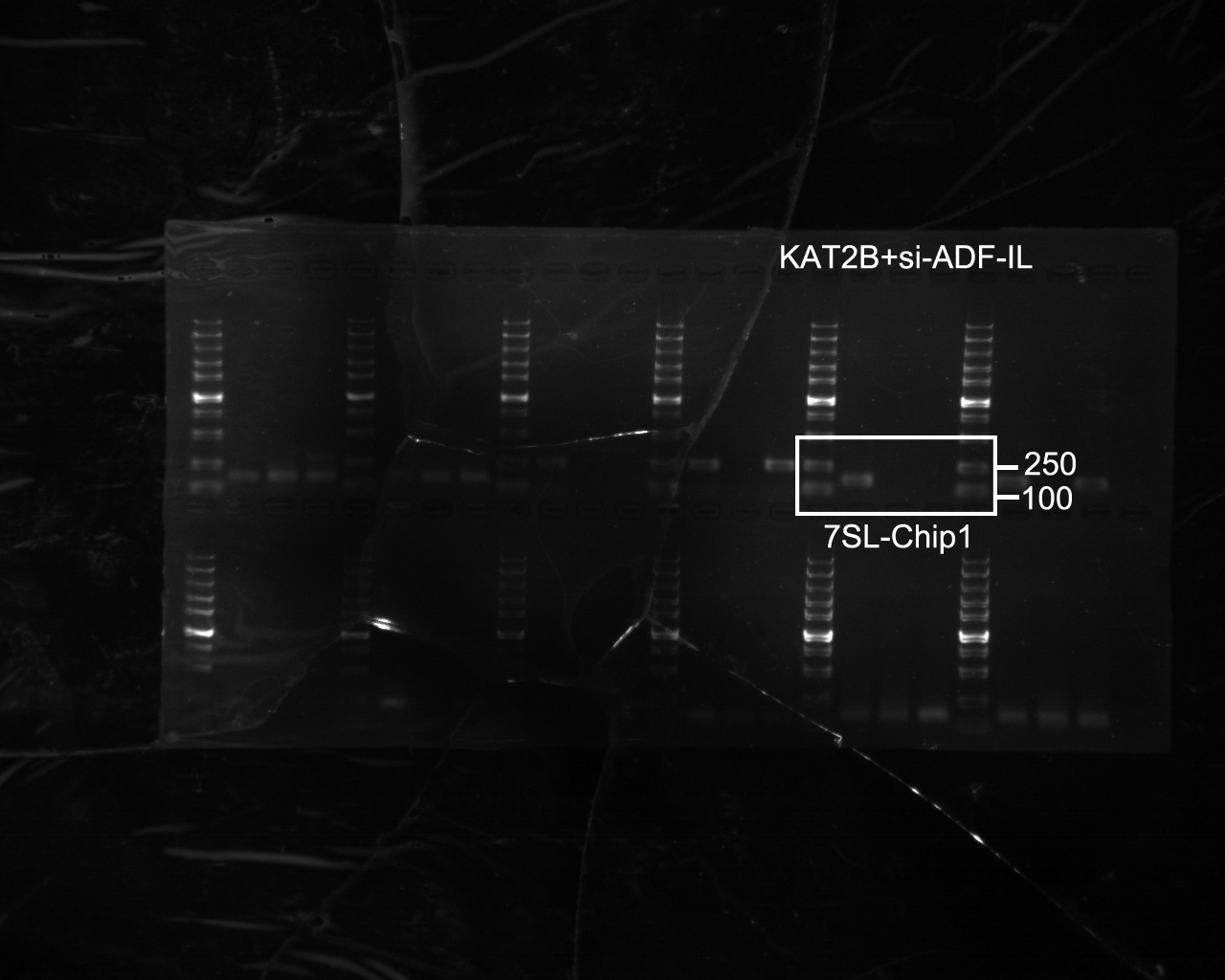

Supplement: Supplementary file 5 — Source data Fig. 3 [file 44319_2025_379_MOESM5_ESM.zip › Figure 3/3D/3C-7SL-Chip1.tif]

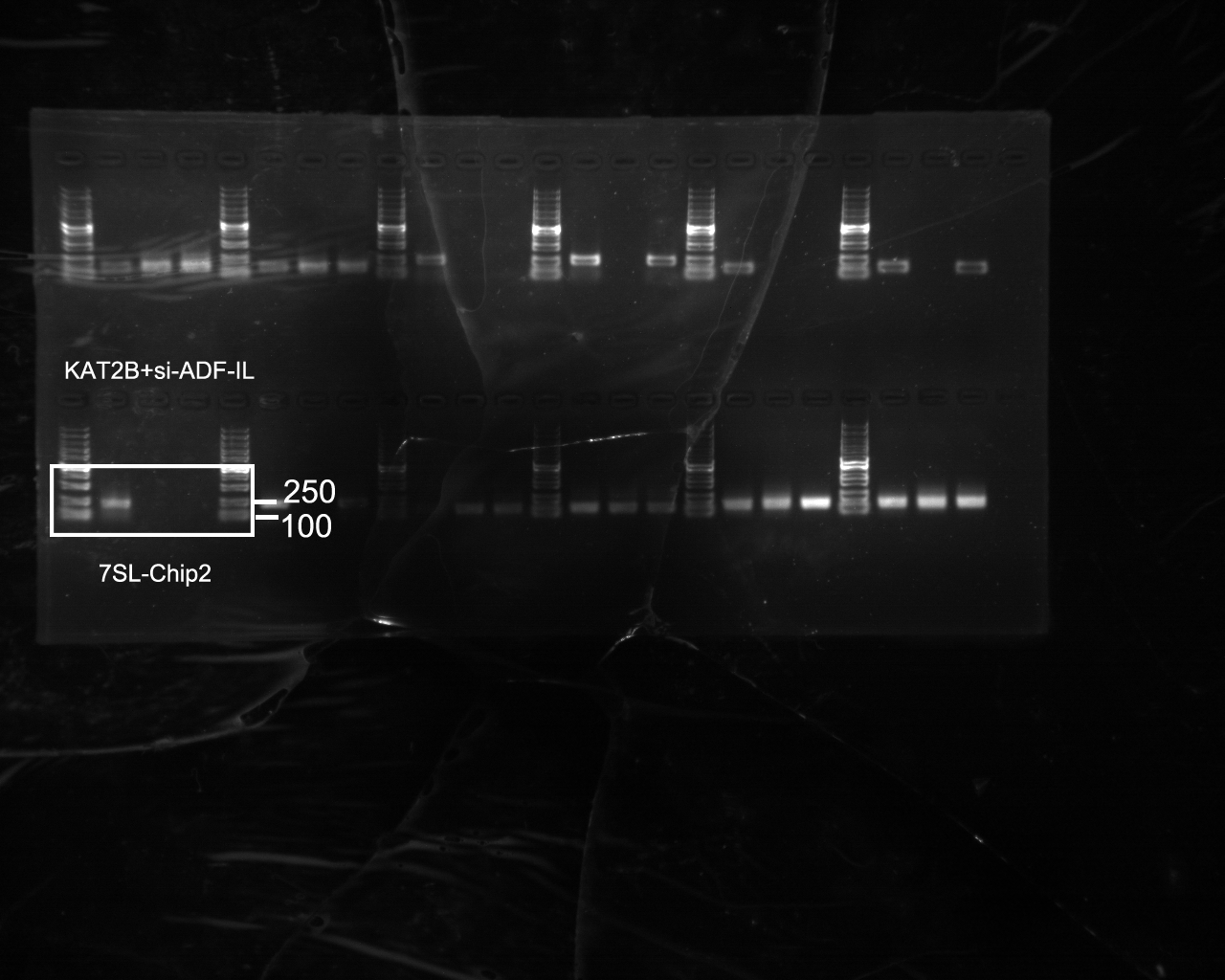

Supplement: Supplementary file 5 — Source data Fig. 3 [file 44319_2025_379_MOESM5_ESM.zip › Figure 3/3D/3C-7SL-Chip2.tif]

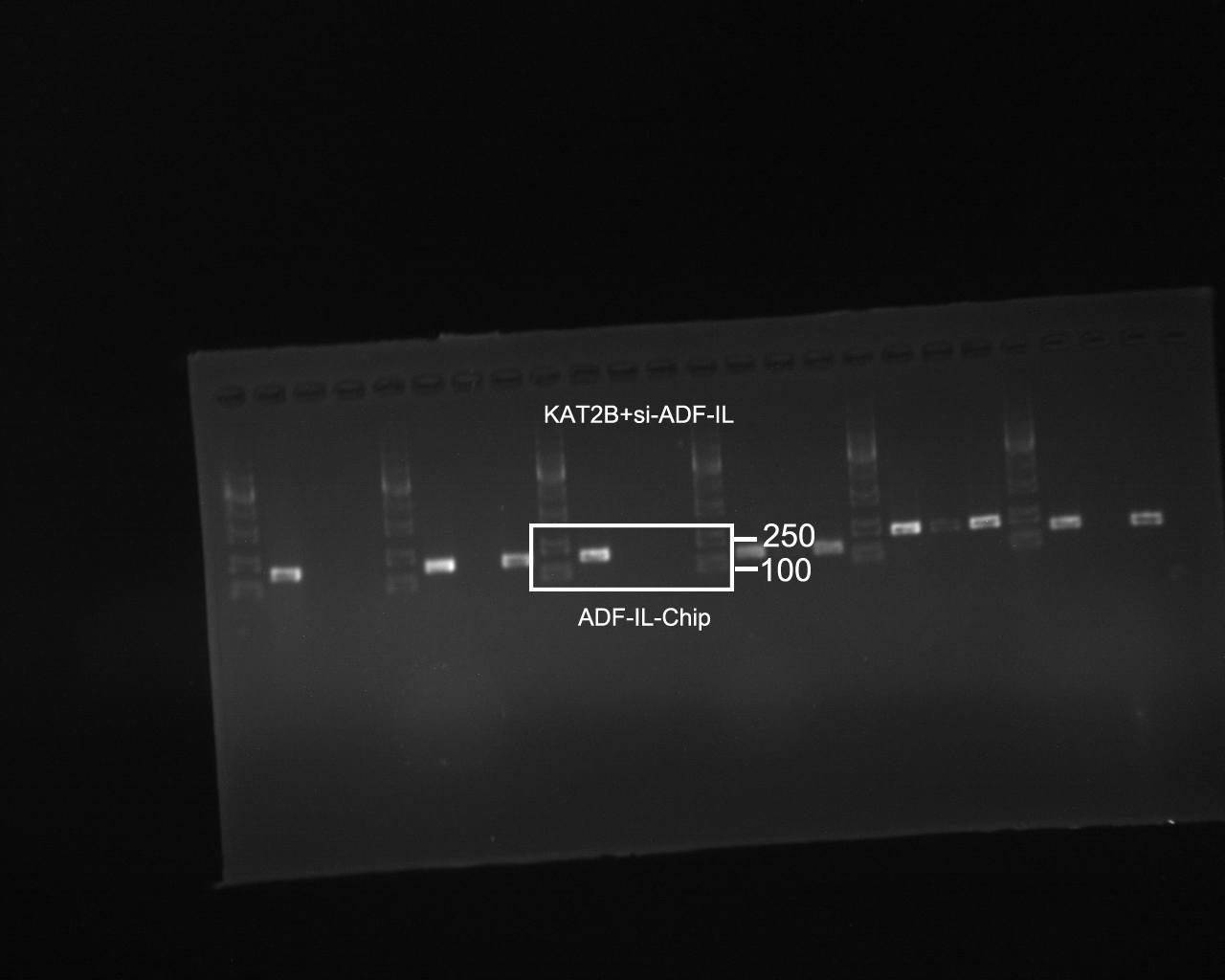

Supplement: Supplementary file 5 — Source data Fig. 3 [file 44319_2025_379_MOESM5_ESM.zip › Figure 3/3D/3D-ADF-IL.tif]

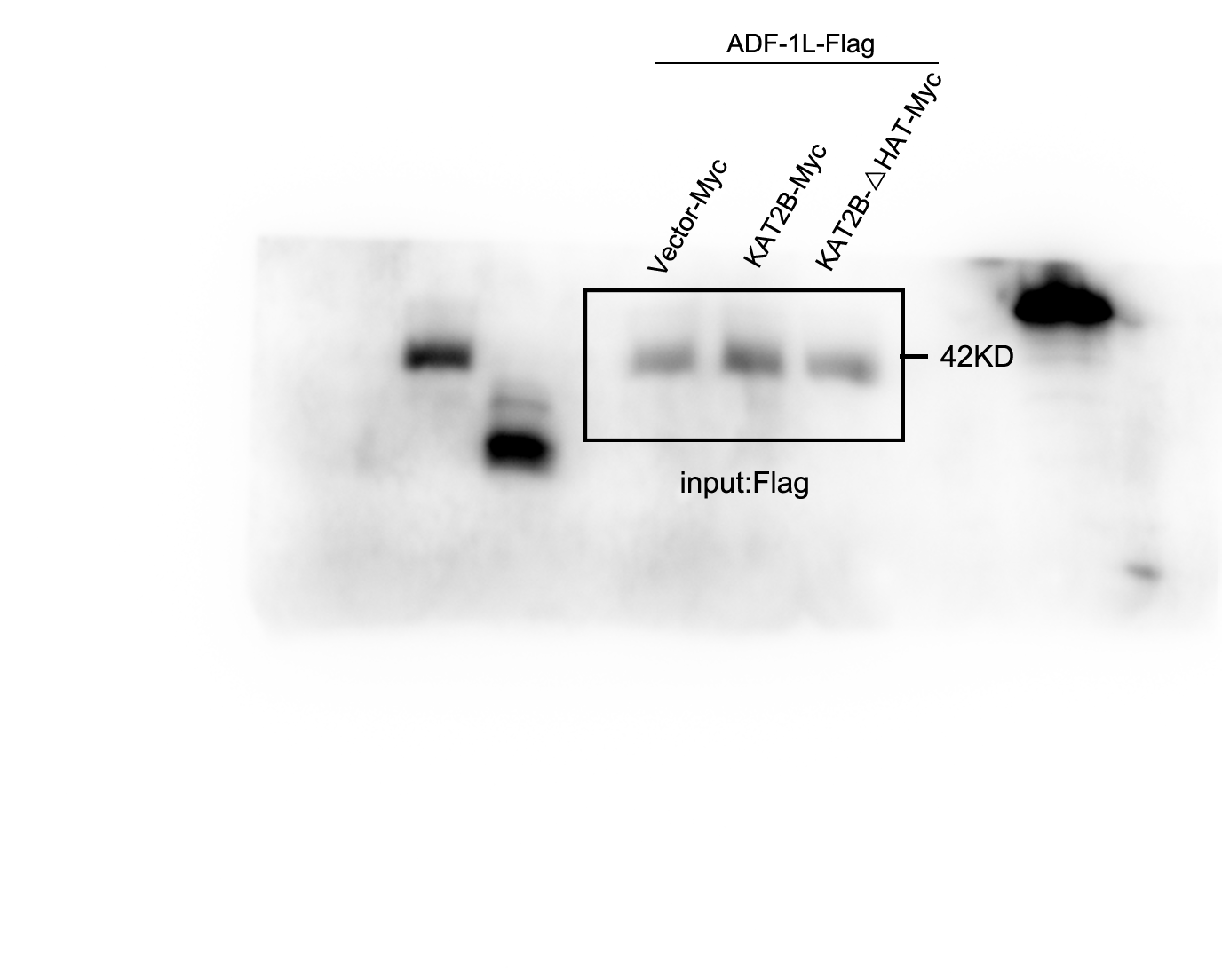

Supplement: Supplementary file 5 — Source data Fig. 3 [file 44319_2025_379_MOESM5_ESM.zip › Figure 3/3F/3F-Input-Flag.tif]

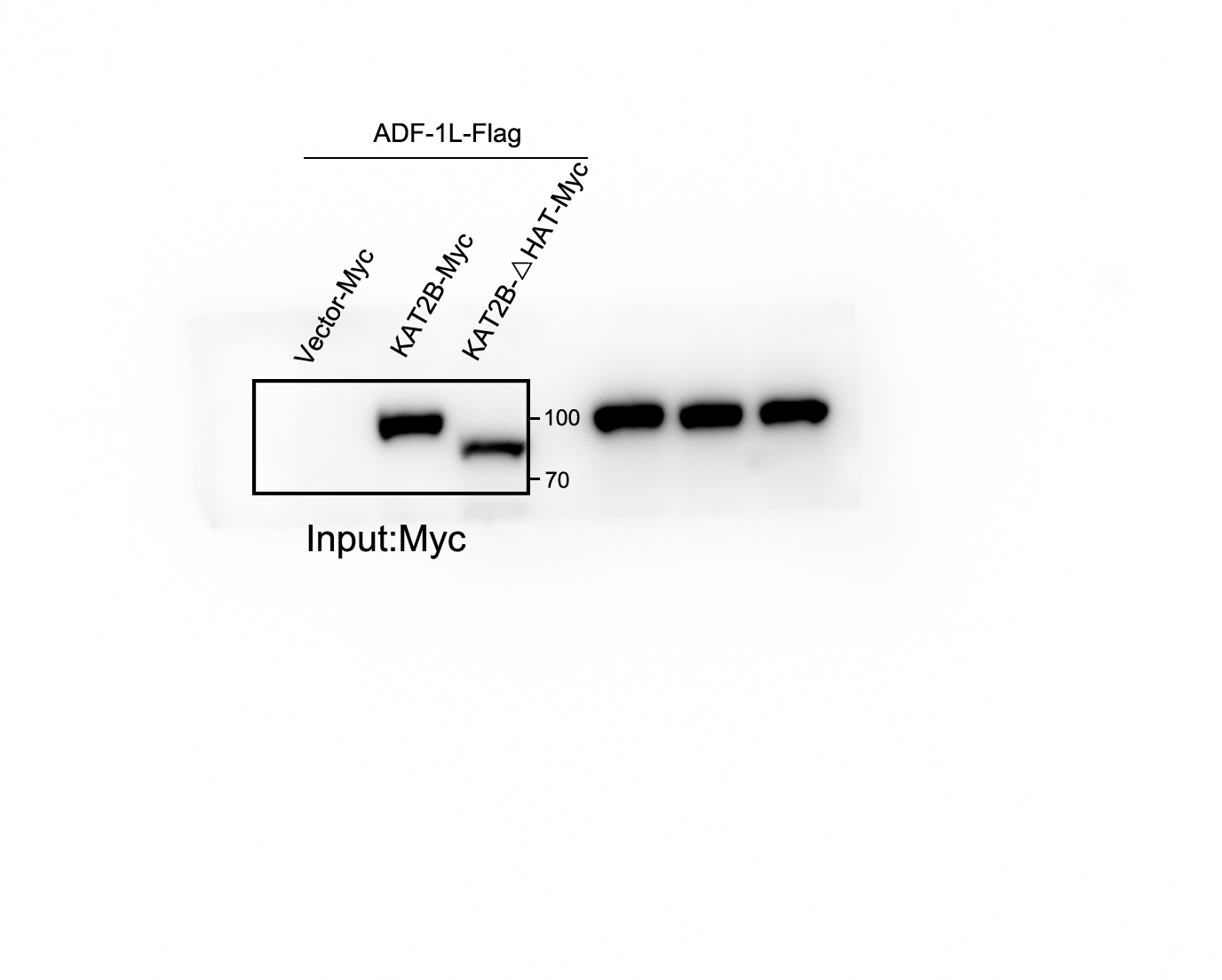

Supplement: Supplementary file 5 — Source data Fig. 3 [file 44319_2025_379_MOESM5_ESM.zip › Figure 3/3F/3F-Input-Myc.tif]

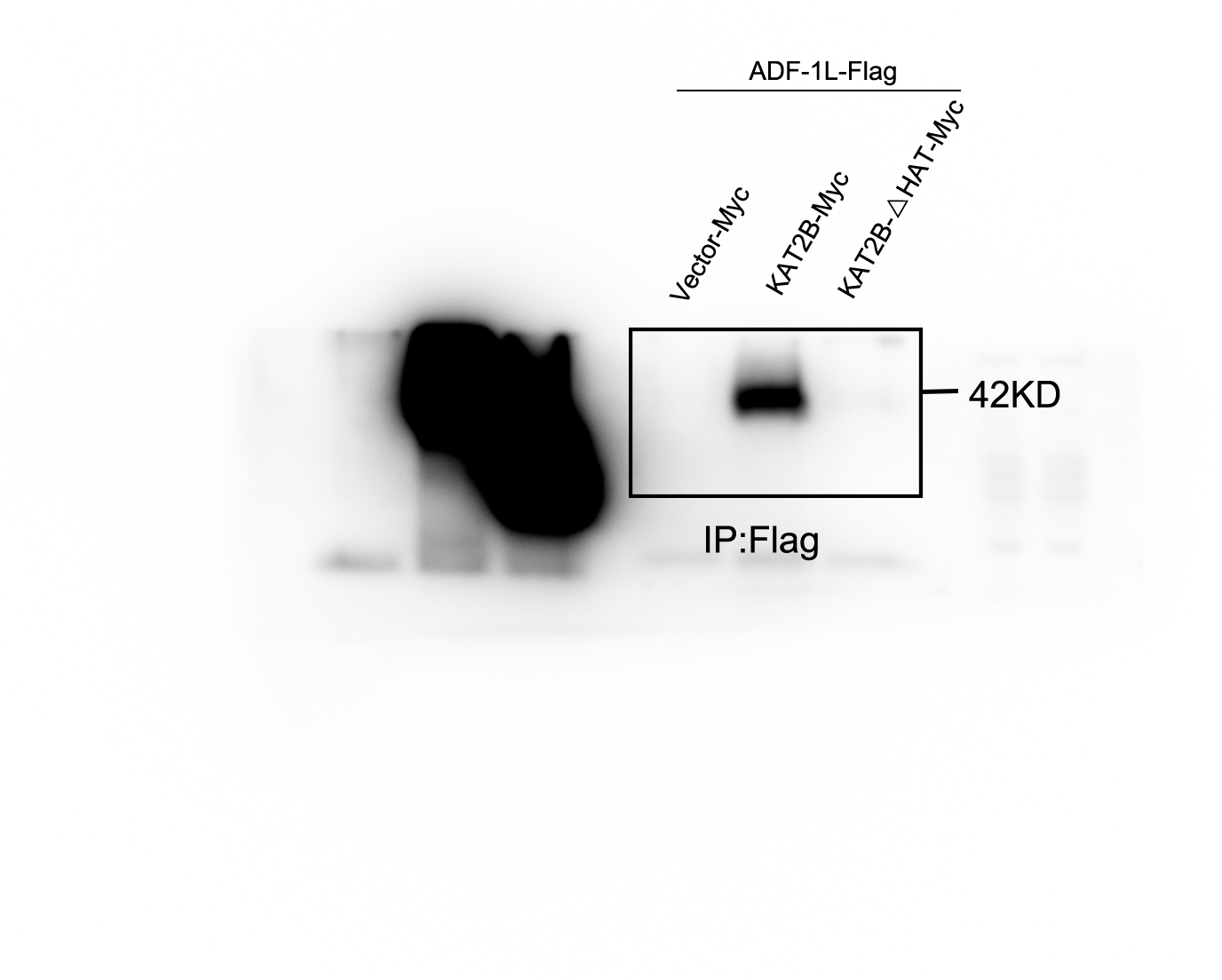

Supplement: Supplementary file 5 — Source data Fig. 3 [file 44319_2025_379_MOESM5_ESM.zip › Figure 3/3F/3F-IP-Flag.tif]

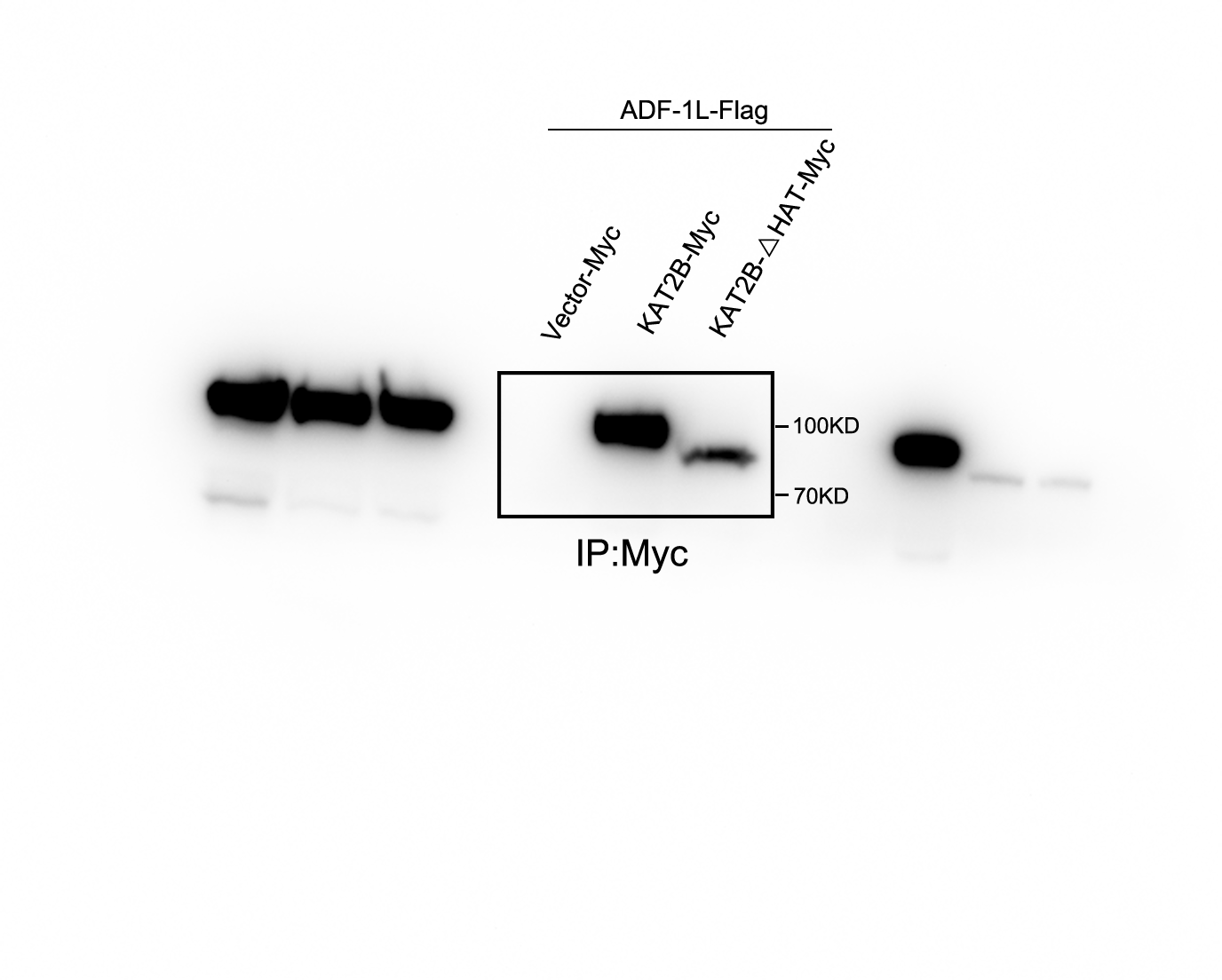

Supplement: Supplementary file 5 — Source data Fig. 3 [file 44319_2025_379_MOESM5_ESM.zip › Figure 3/3F/3F-IP-Myc.tif]

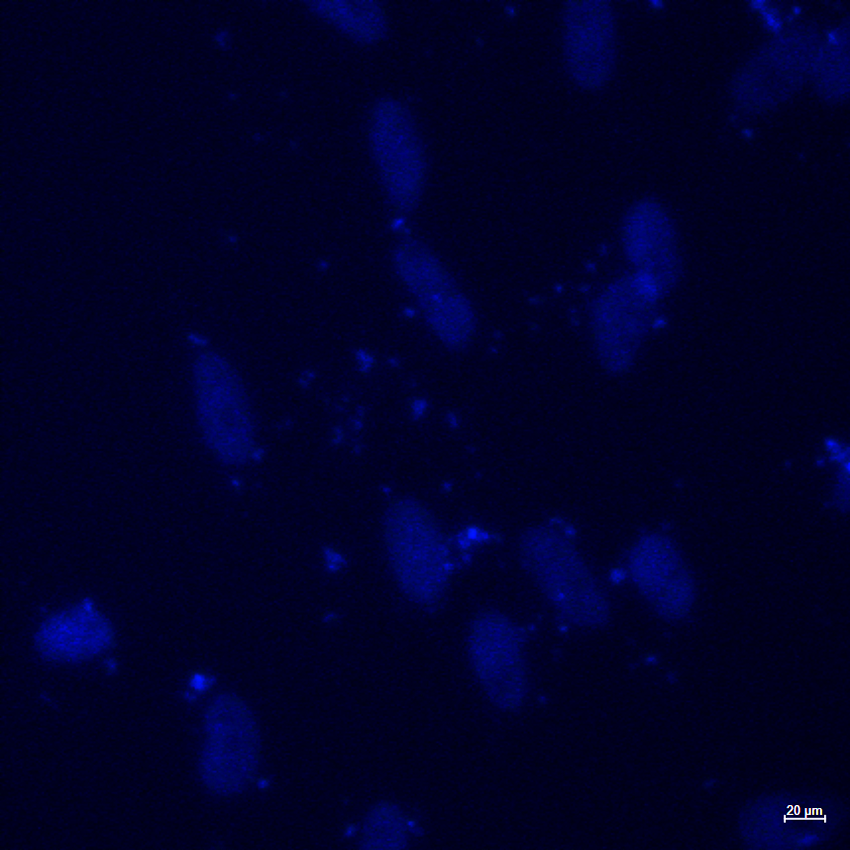

Supplement: Supplementary file 5 — Source data Fig. 3 [file 44319_2025_379_MOESM5_ESM.zip › Figure 3/3G/3G-ADF-IL+KAT2B-DAPI.tif]

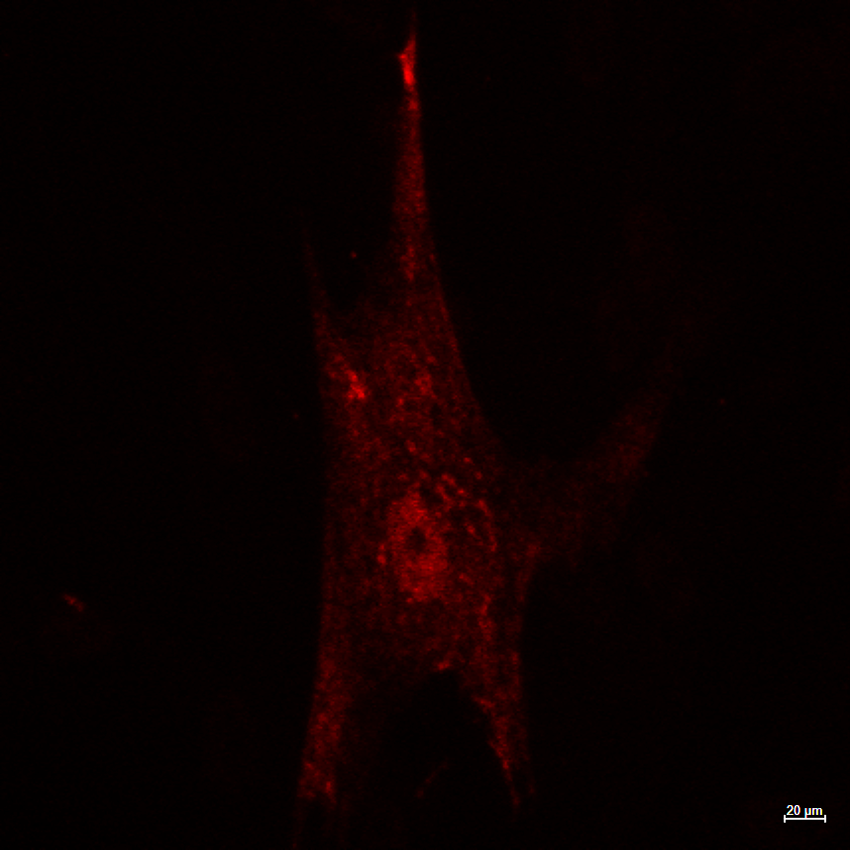

Supplement: Supplementary file 5 — Source data Fig. 3 [file 44319_2025_379_MOESM5_ESM.zip › Figure 3/3G/3G-ADF-IL+KAT2B-Flag.tif]

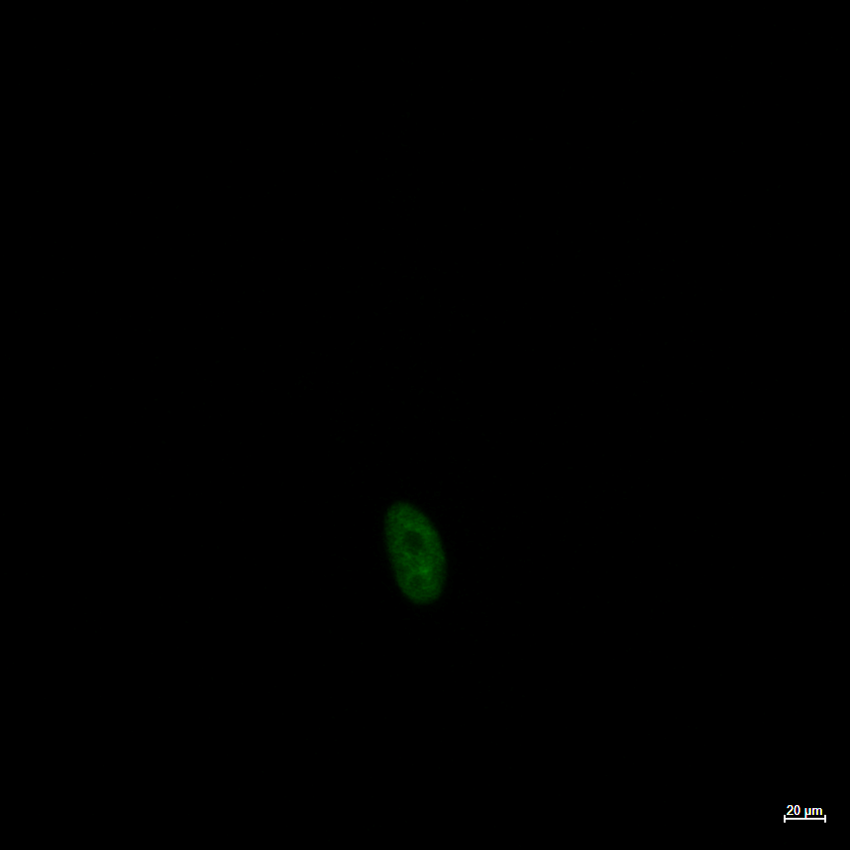

Supplement: Supplementary file 5 — Source data Fig. 3 [file 44319_2025_379_MOESM5_ESM.zip › Figure 3/3G/3G-ADF-IL+KAT2B-Myc.tif]

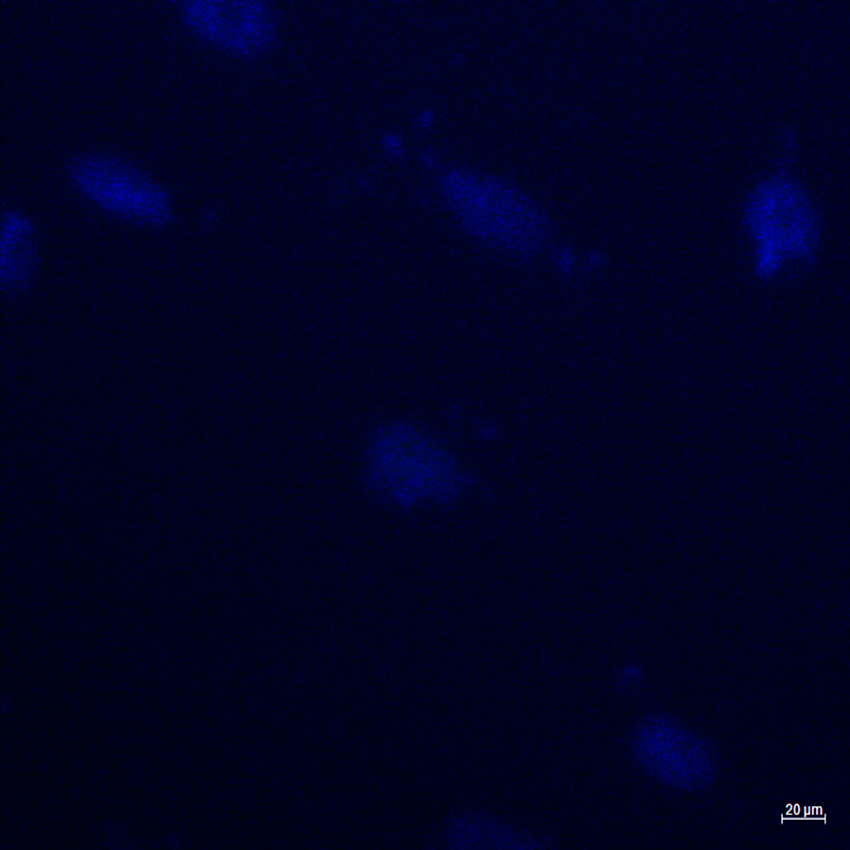

Supplement: Supplementary file 5 — Source data Fig. 3 [file 44319_2025_379_MOESM5_ESM.zip › Figure 3/3G/3G-ADF-IL+KAT2B-△HAT-DAPI.tif]

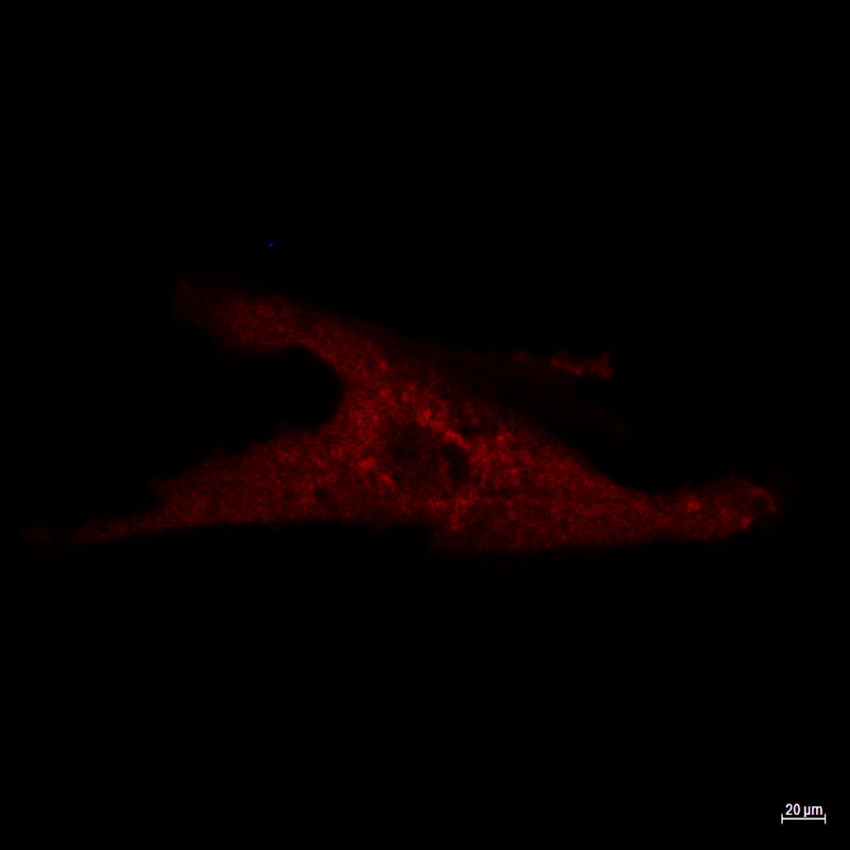

Supplement: Supplementary file 5 — Source data Fig. 3 [file 44319_2025_379_MOESM5_ESM.zip › Figure 3/3G/3G-ADF-IL+KAT2B-△HAT-Flag.tif]

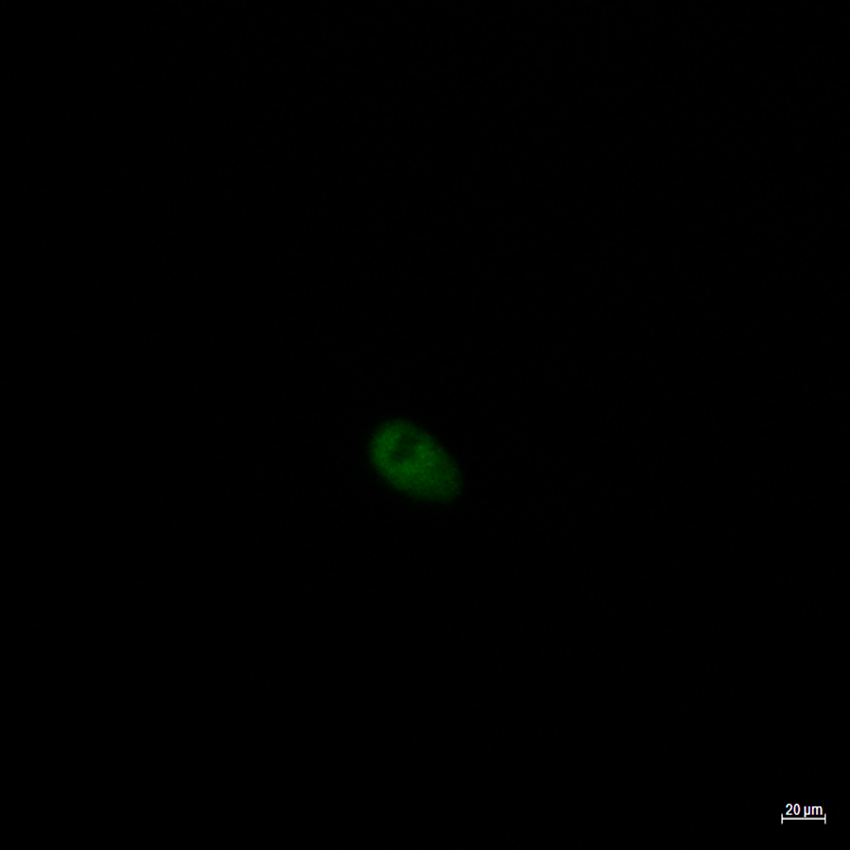

Supplement: Supplementary file 5 — Source data Fig. 3 [file 44319_2025_379_MOESM5_ESM.zip › Figure 3/3G/3G-ADF-IL+KAT2B-△HAT-Myc.tif]

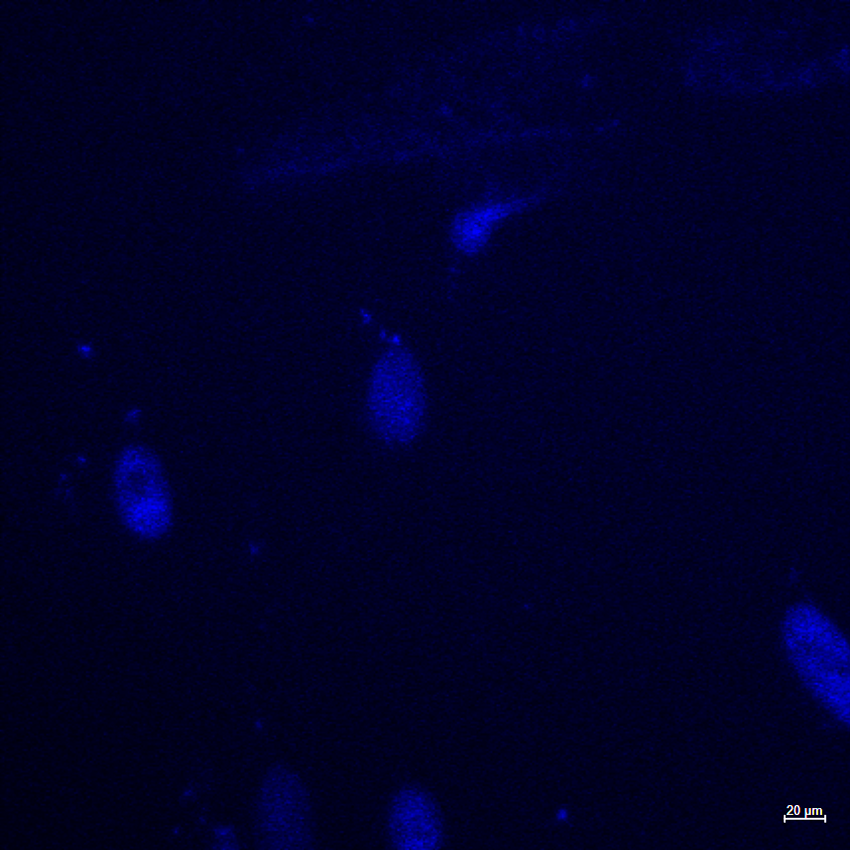

Supplement: Supplementary file 5 — Source data Fig. 3 [file 44319_2025_379_MOESM5_ESM.zip › Figure 3/3G/3G-ADF-IL-DAPI.tif]

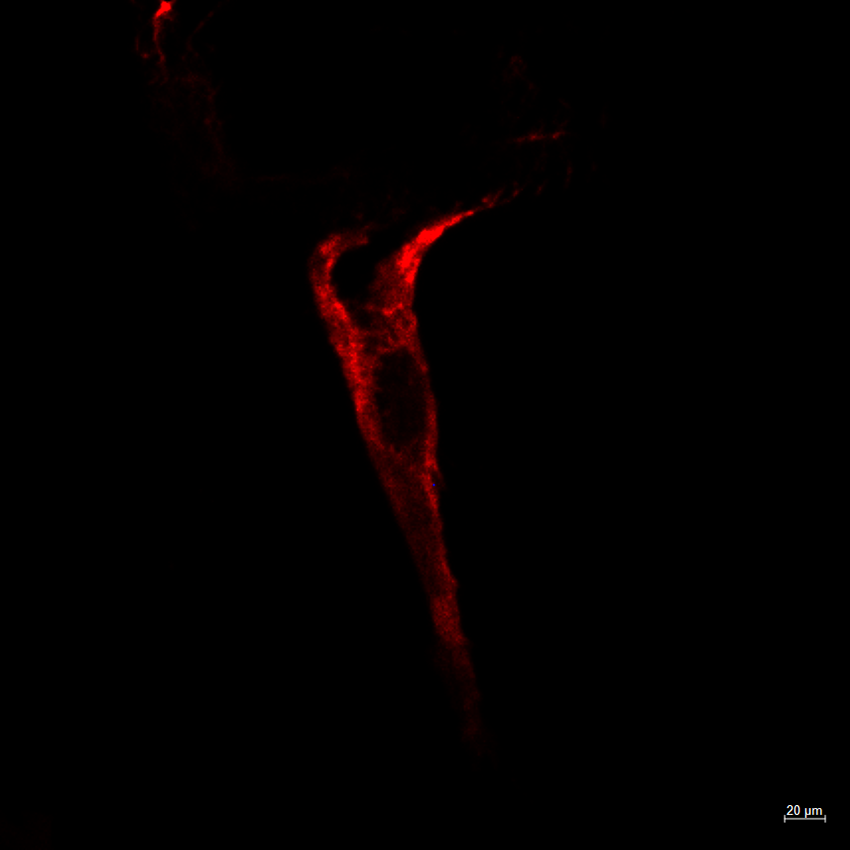

Supplement: Supplementary file 5 — Source data Fig. 3 [file 44319_2025_379_MOESM5_ESM.zip › Figure 3/3G/3G-ADF-IL-Flag.tif]

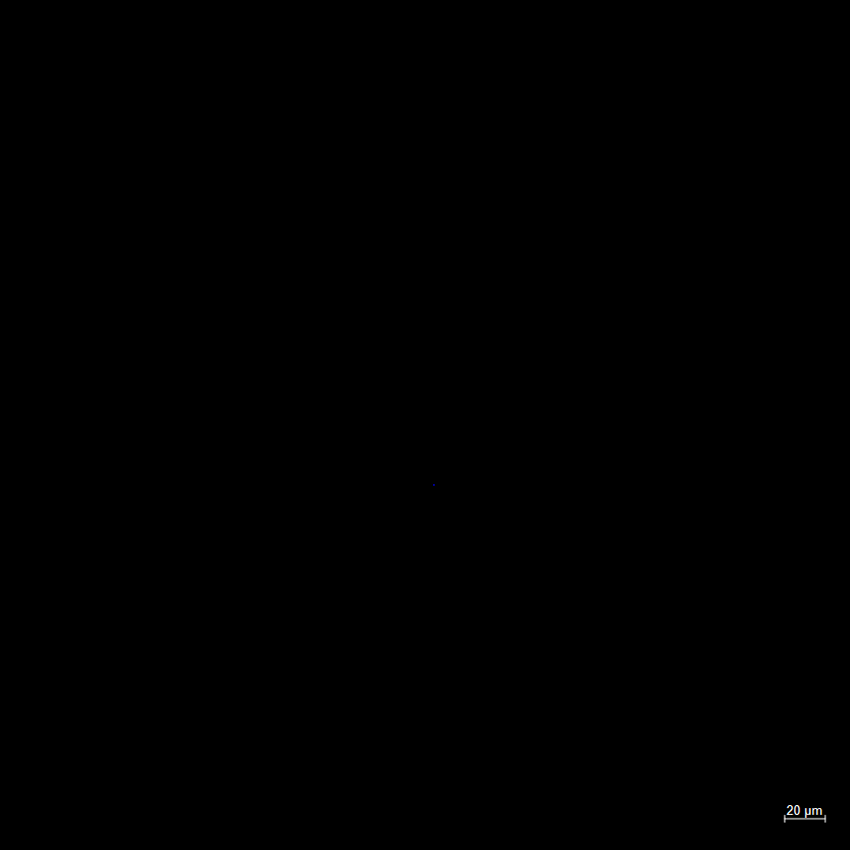

Supplement: Supplementary file 5 — Source data Fig. 3 [file 44319_2025_379_MOESM5_ESM.zip › Figure 3/3G/3G-ADF-IL-Myc.tif]

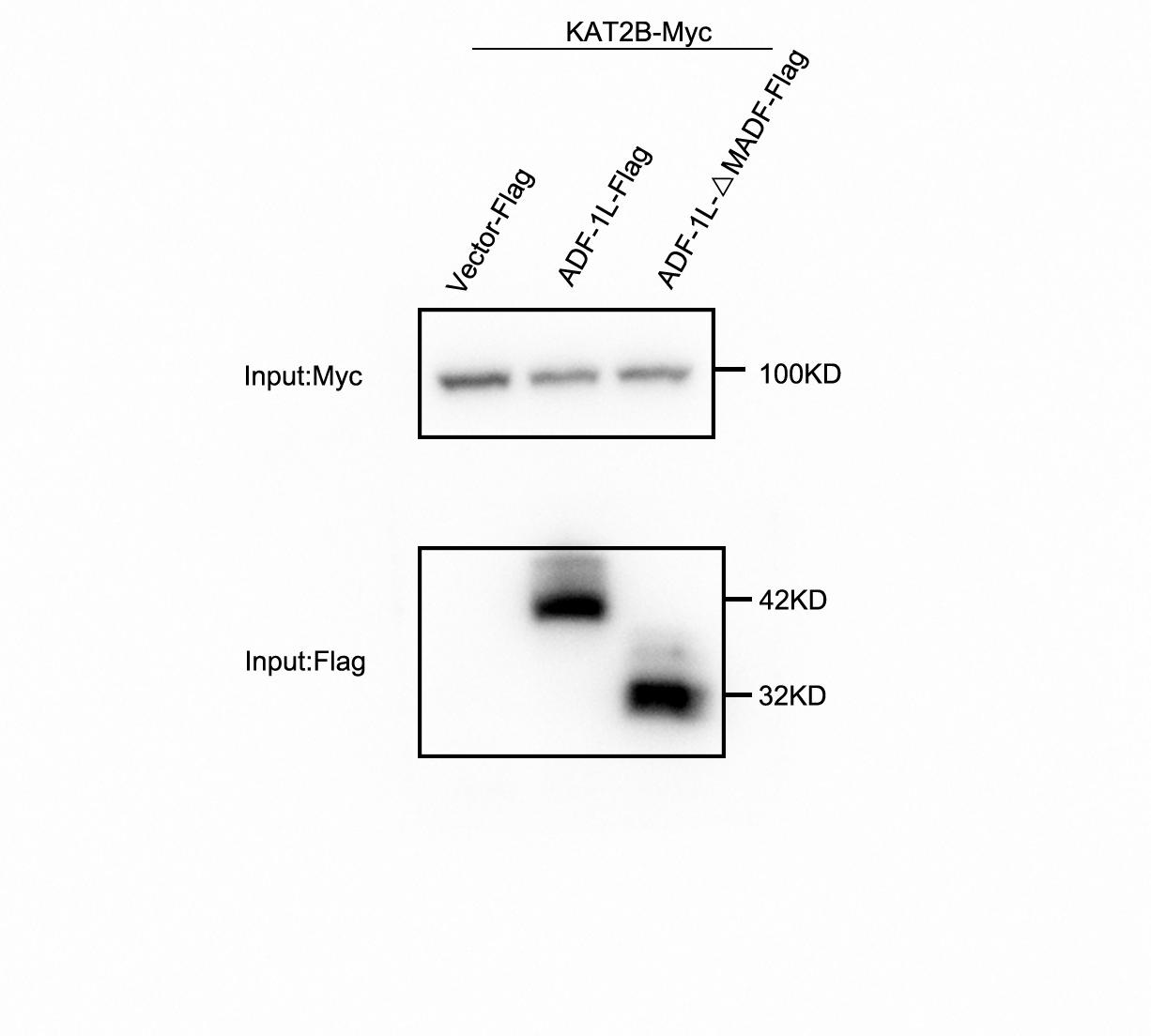

Supplement: Supplementary file 5 — Source data Fig. 3 [file 44319_2025_379_MOESM5_ESM.zip › Figure 3/3K/3K-input.tif]

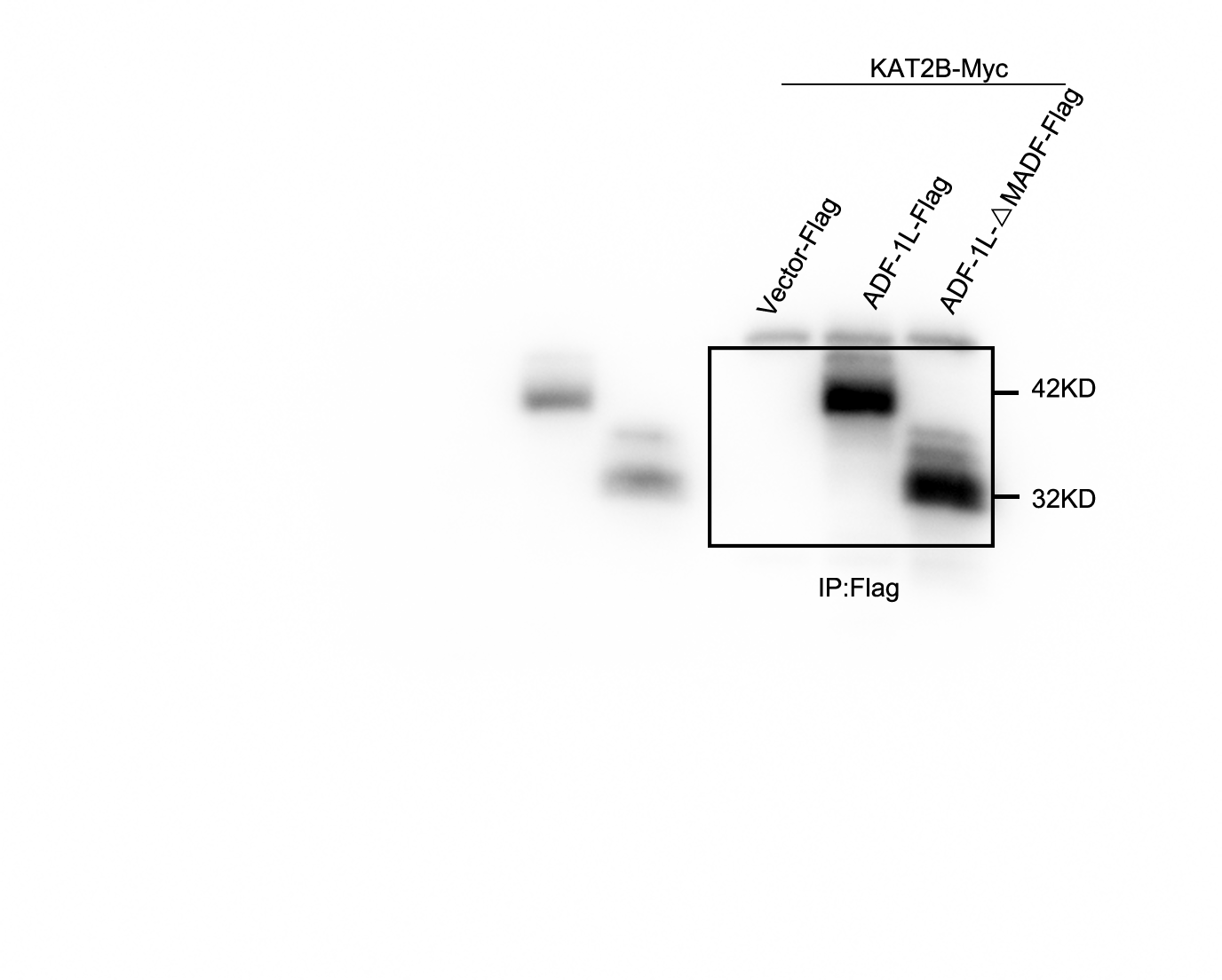

Supplement: Supplementary file 5 — Source data Fig. 3 [file 44319_2025_379_MOESM5_ESM.zip › Figure 3/3K/3K-IP-Flag.tif]

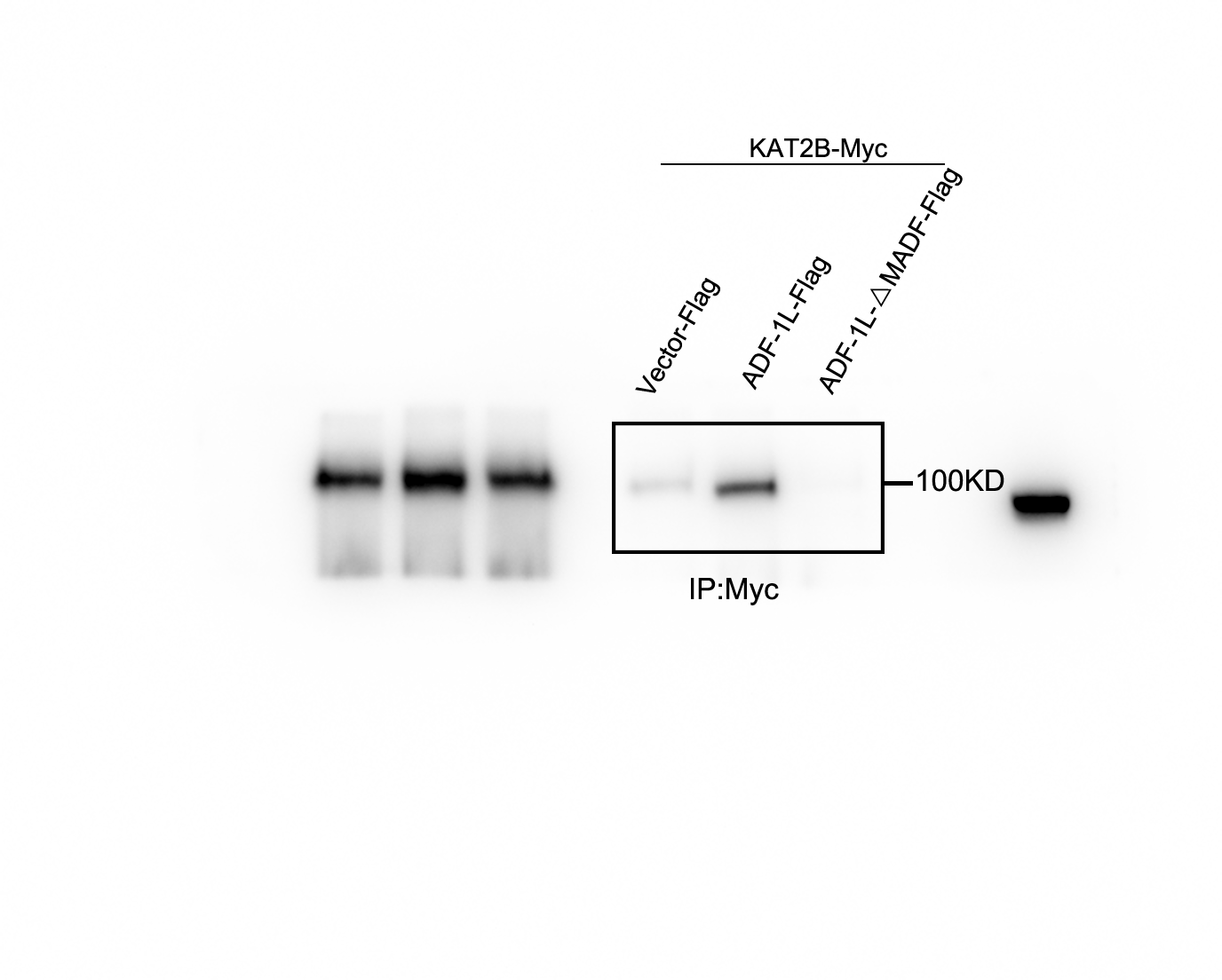

Supplement: Supplementary file 5 — Source data Fig. 3 [file 44319_2025_379_MOESM5_ESM.zip › Figure 3/3K/3K-IP-Myc.tif]

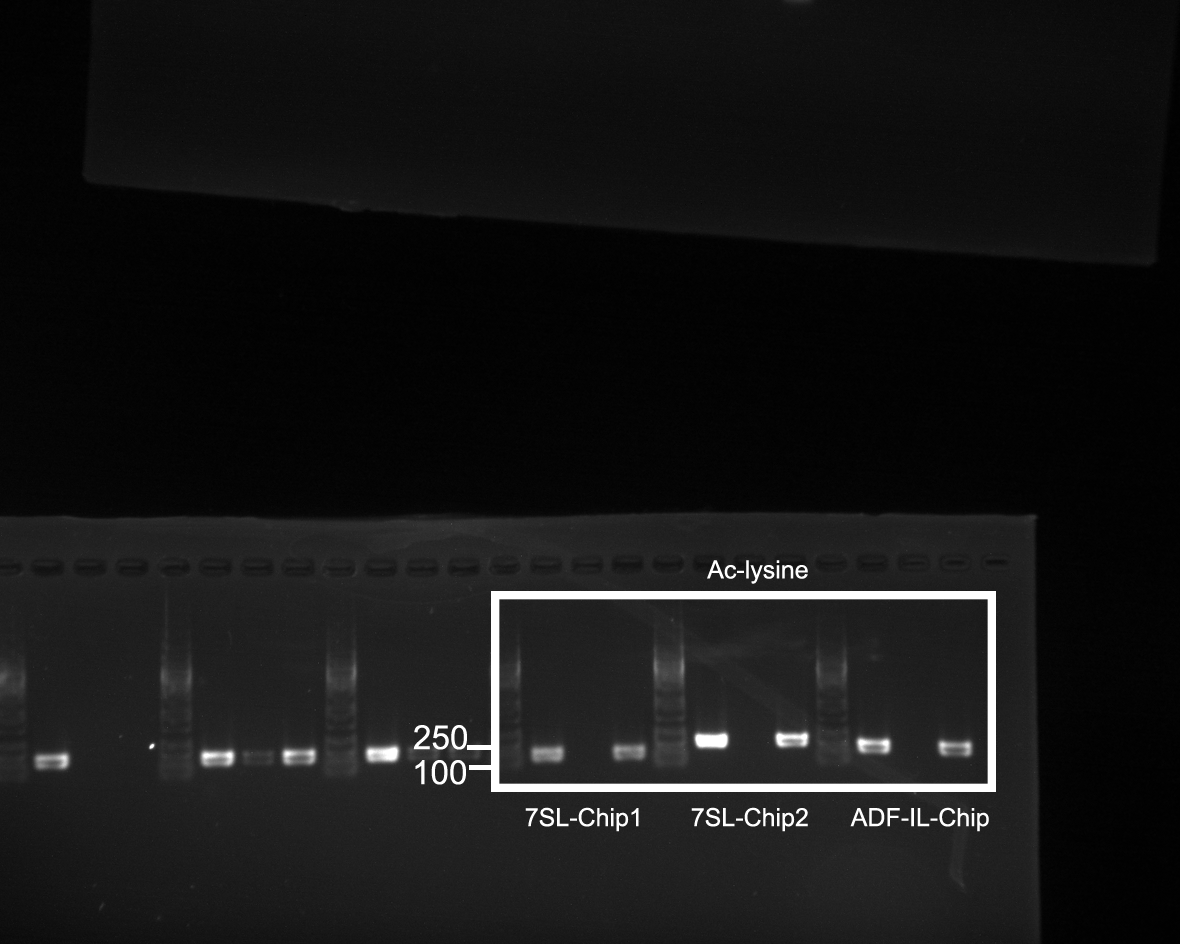

Supplement: Supplementary file 5 — Source data Fig. 3 [file 44319_2025_379_MOESM5_ESM.zip › Figure 3/3M/3M.tif]

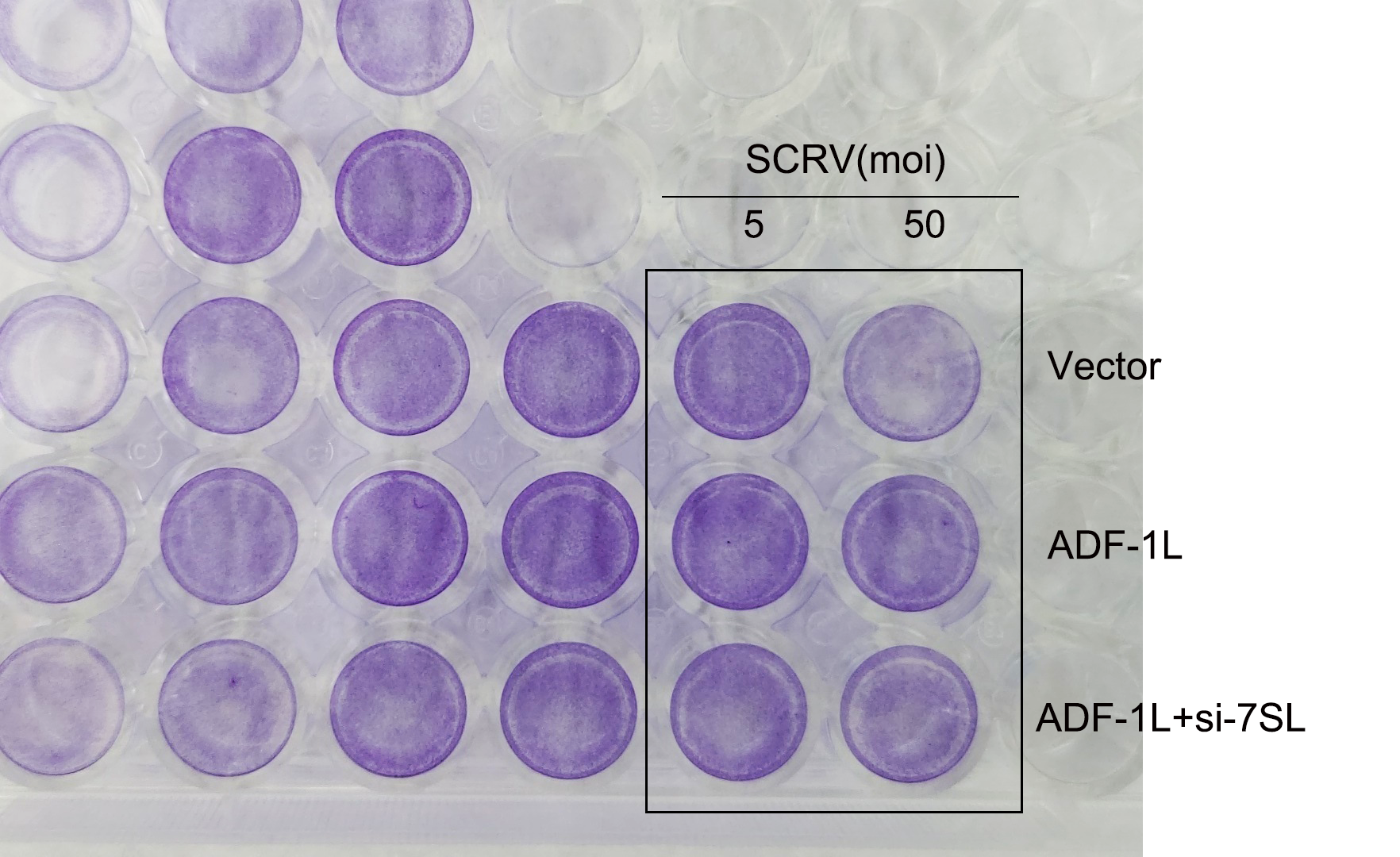

Supplement: Supplementary file 6 — Source data Fig. 4 [file 44319_2025_379_MOESM6_ESM.zip › Figure 4/4H/4H.tif]

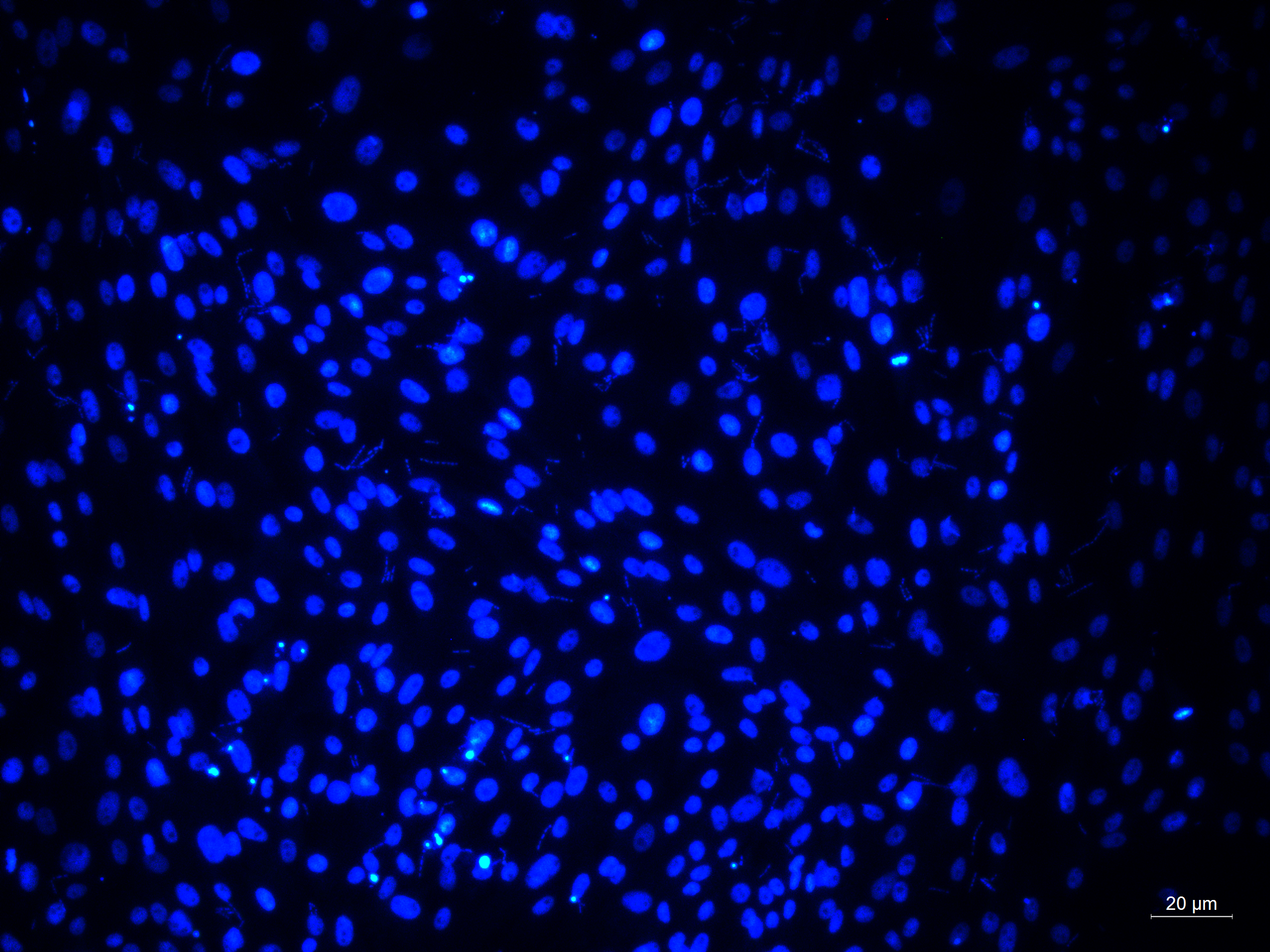

Supplement: Supplementary file 6 — Source data Fig. 4 [file 44319_2025_379_MOESM6_ESM.zip › Figure 4/4J/4J-ADF-IL+si-7SL-DAPI.tif]

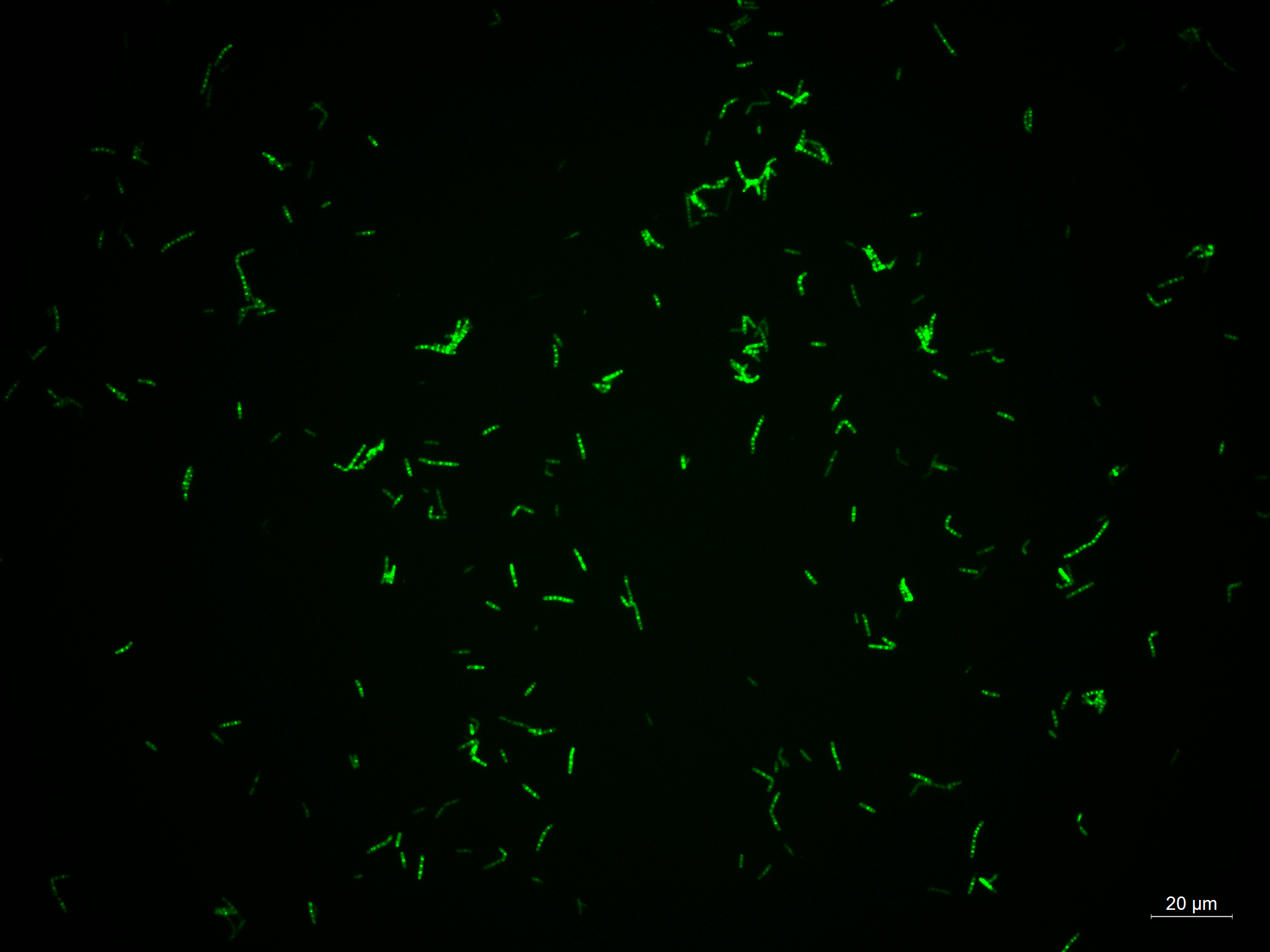

Supplement: Supplementary file 6 — Source data Fig. 4 [file 44319_2025_379_MOESM6_ESM.zip › Figure 4/4J/4J-ADF-IL+si-7SL-FITC.tif]

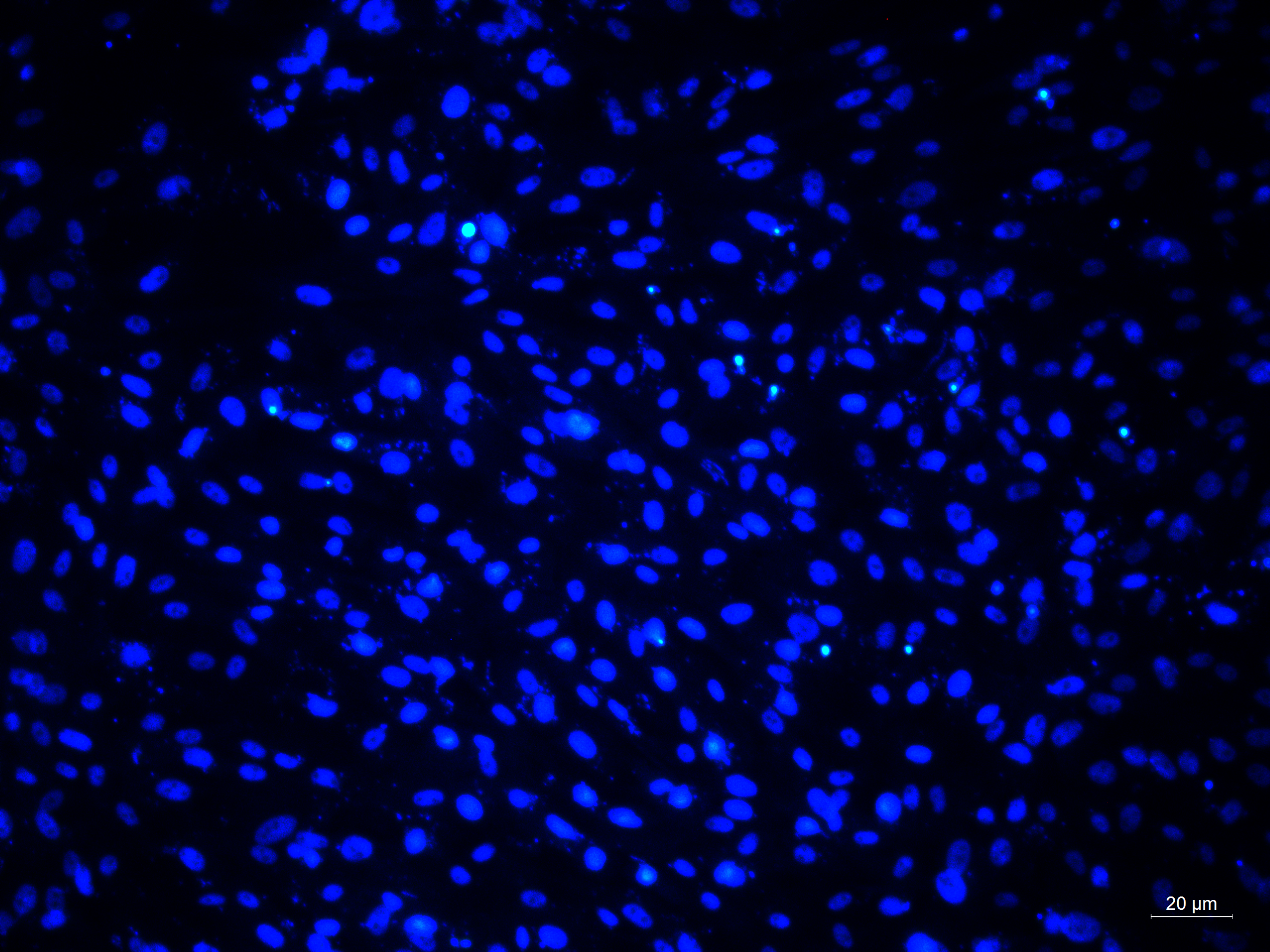

Supplement: Supplementary file 6 — Source data Fig. 4 [file 44319_2025_379_MOESM6_ESM.zip › Figure 4/4J/4J-ADF-IL-DAPI.tif]

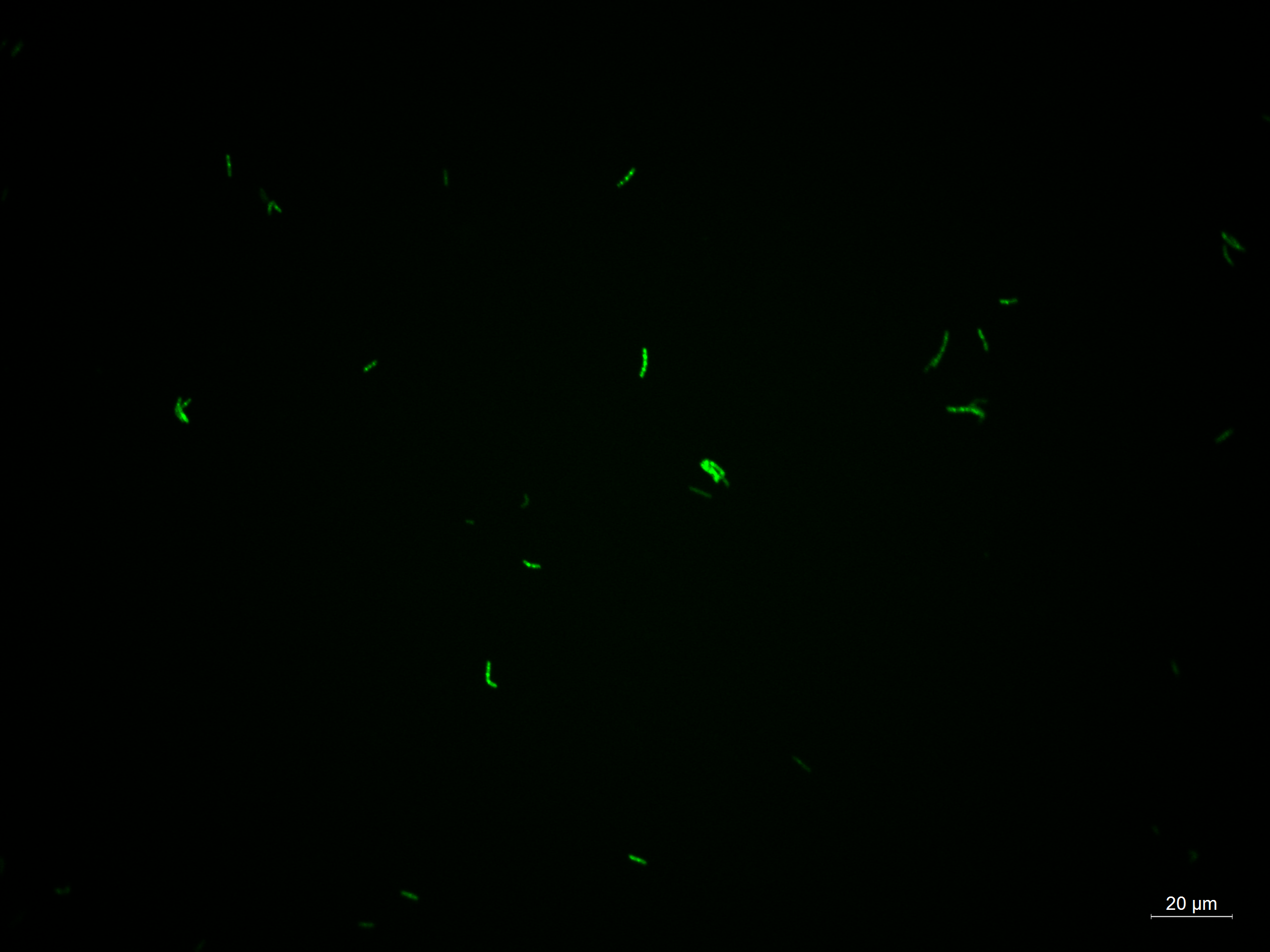

Supplement: Supplementary file 6 — Source data Fig. 4 [file 44319_2025_379_MOESM6_ESM.zip › Figure 4/4J/4J-ADF-IL-FITC.tif]

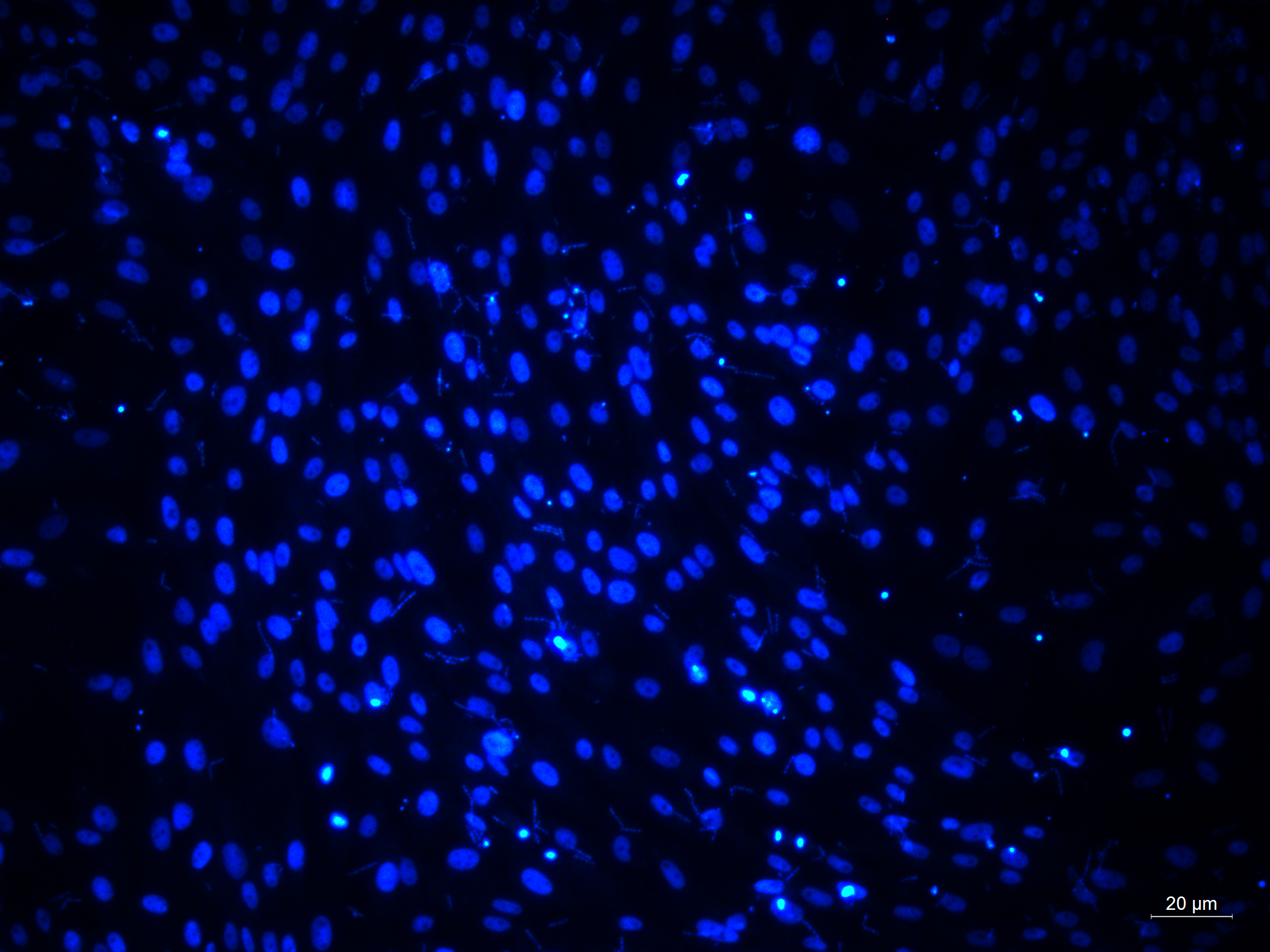

Supplement: Supplementary file 6 — Source data Fig. 4 [file 44319_2025_379_MOESM6_ESM.zip › Figure 4/4J/4J-vector-DAPI.tif]

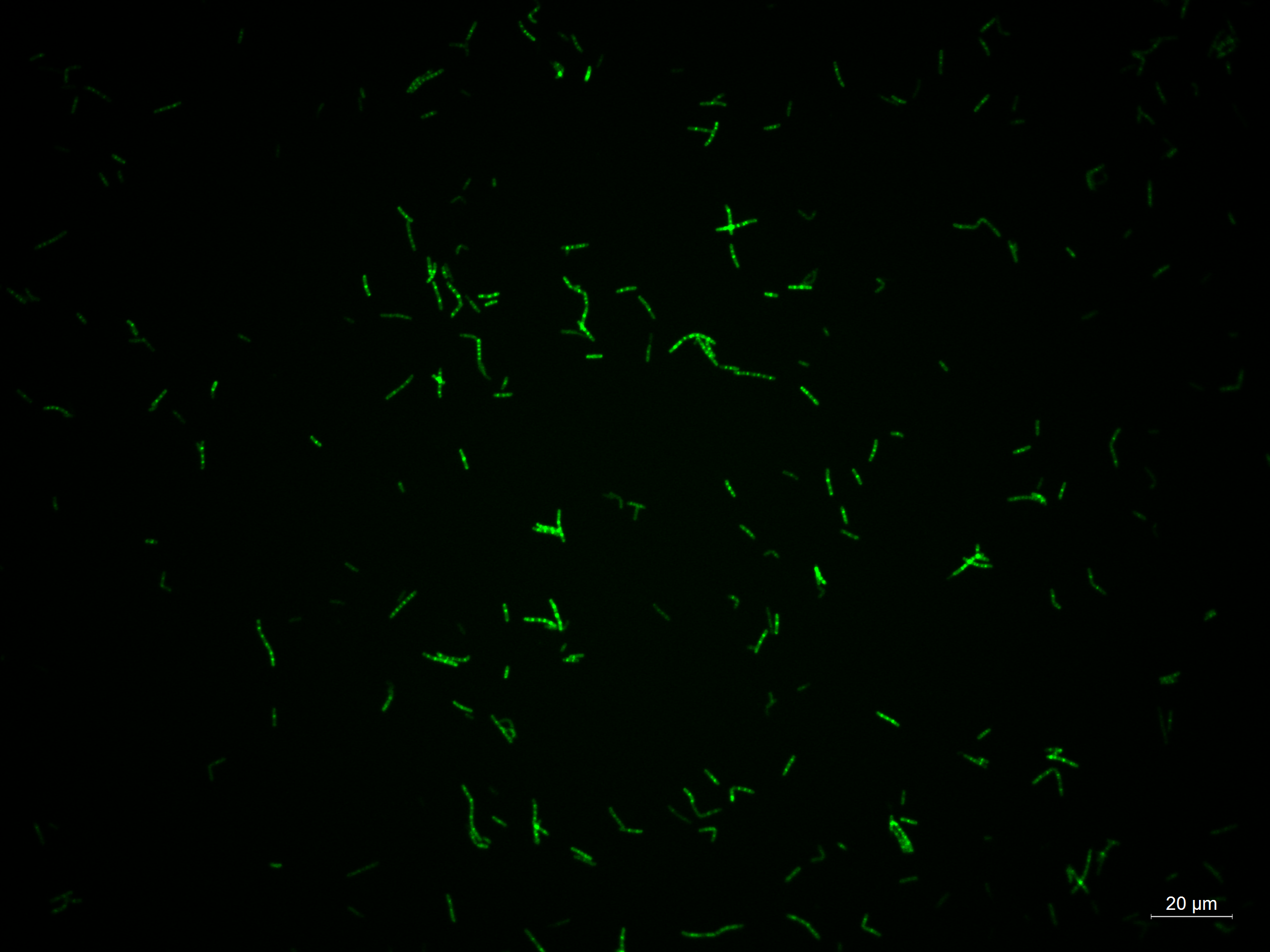

Supplement: Supplementary file 6 — Source data Fig. 4 [file 44319_2025_379_MOESM6_ESM.zip › Figure 4/4J/4J-vector-FITC.tif]

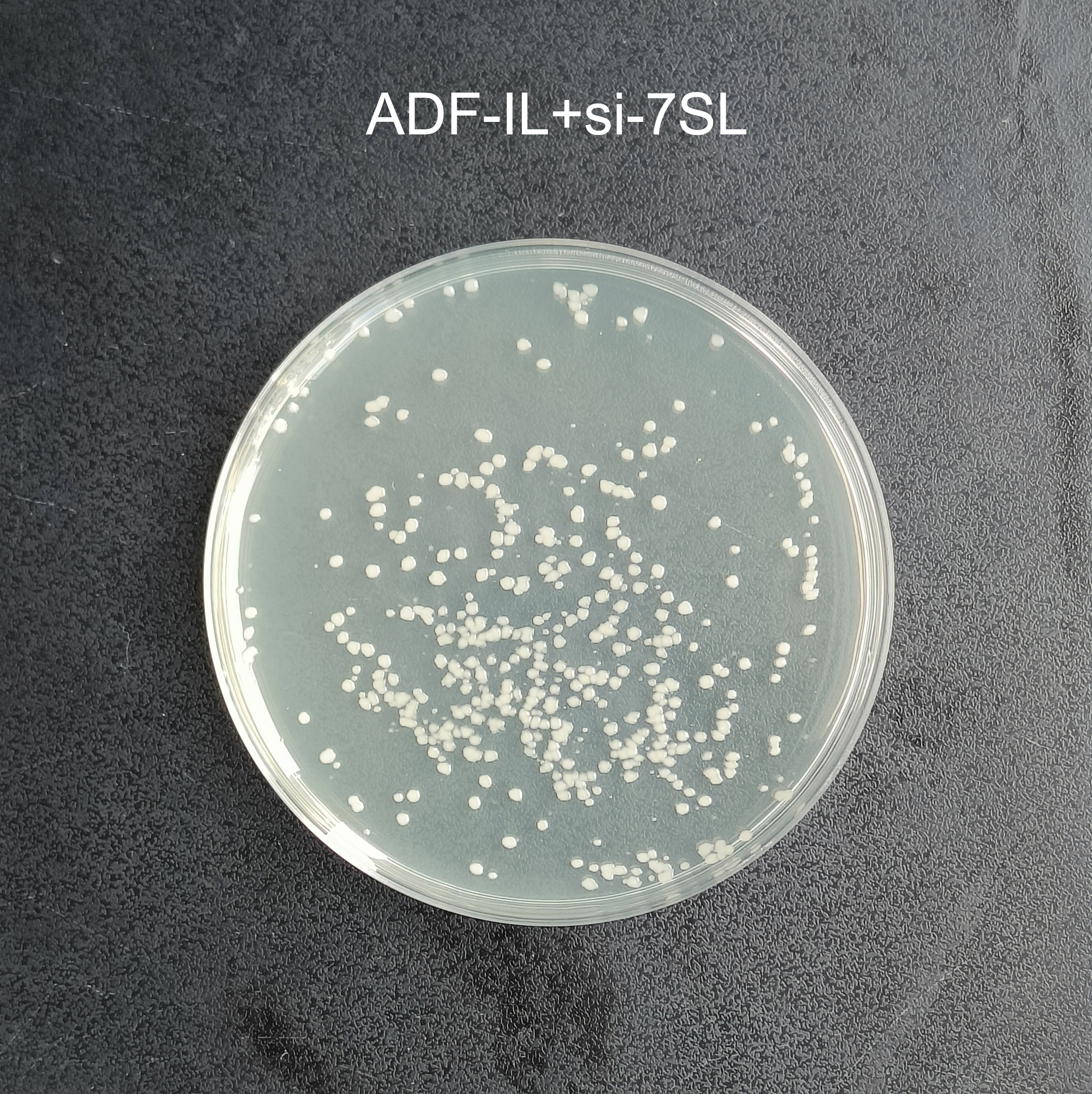

Supplement: Supplementary file 6 — Source data Fig. 4 [file 44319_2025_379_MOESM6_ESM.zip › Figure 4/4K/4K-ADF-IL+si-7SL.jpg]

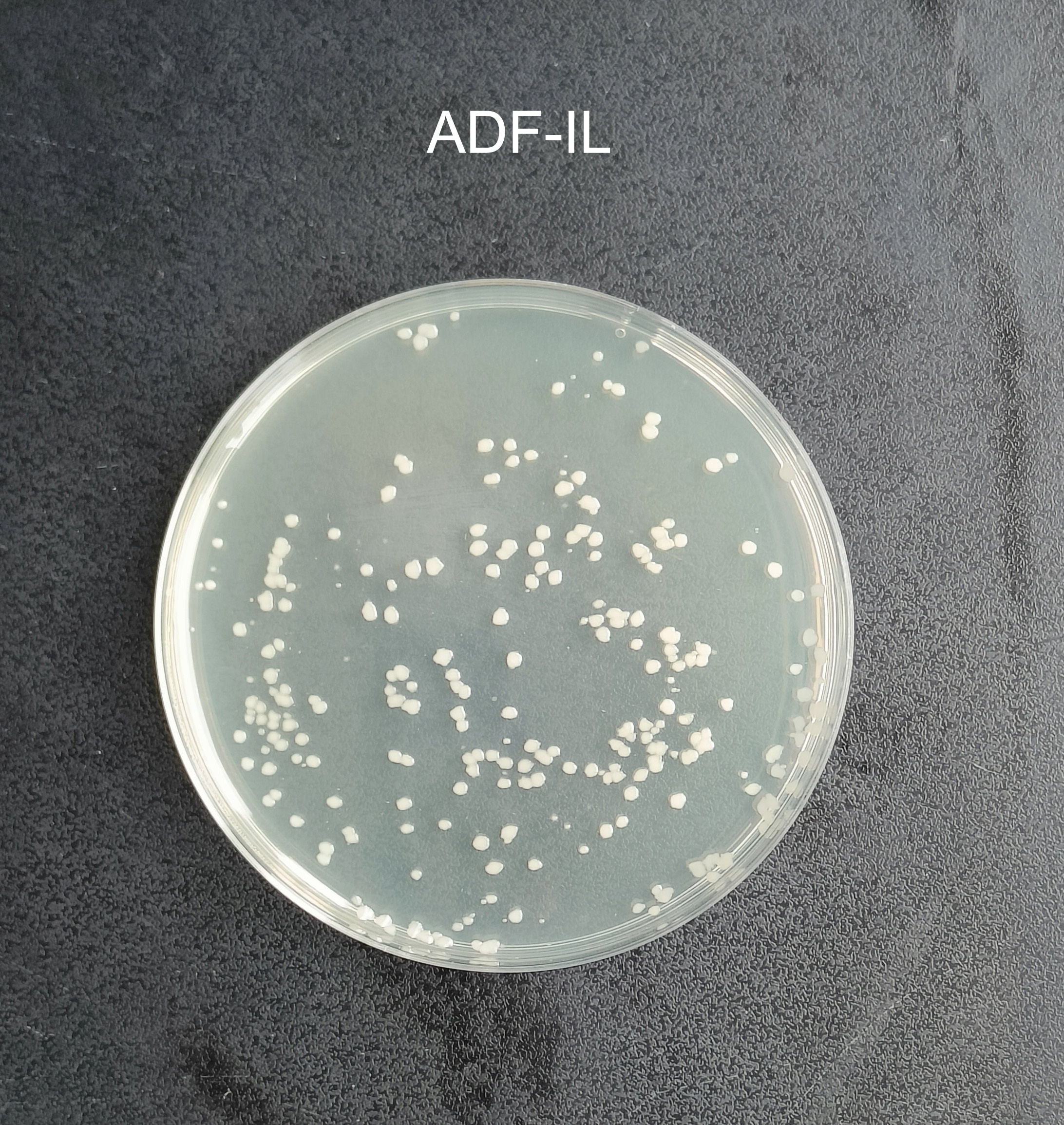

Supplement: Supplementary file 6 — Source data Fig. 4 [file 44319_2025_379_MOESM6_ESM.zip › Figure 4/4K/4K-ADF-IL.jpg]

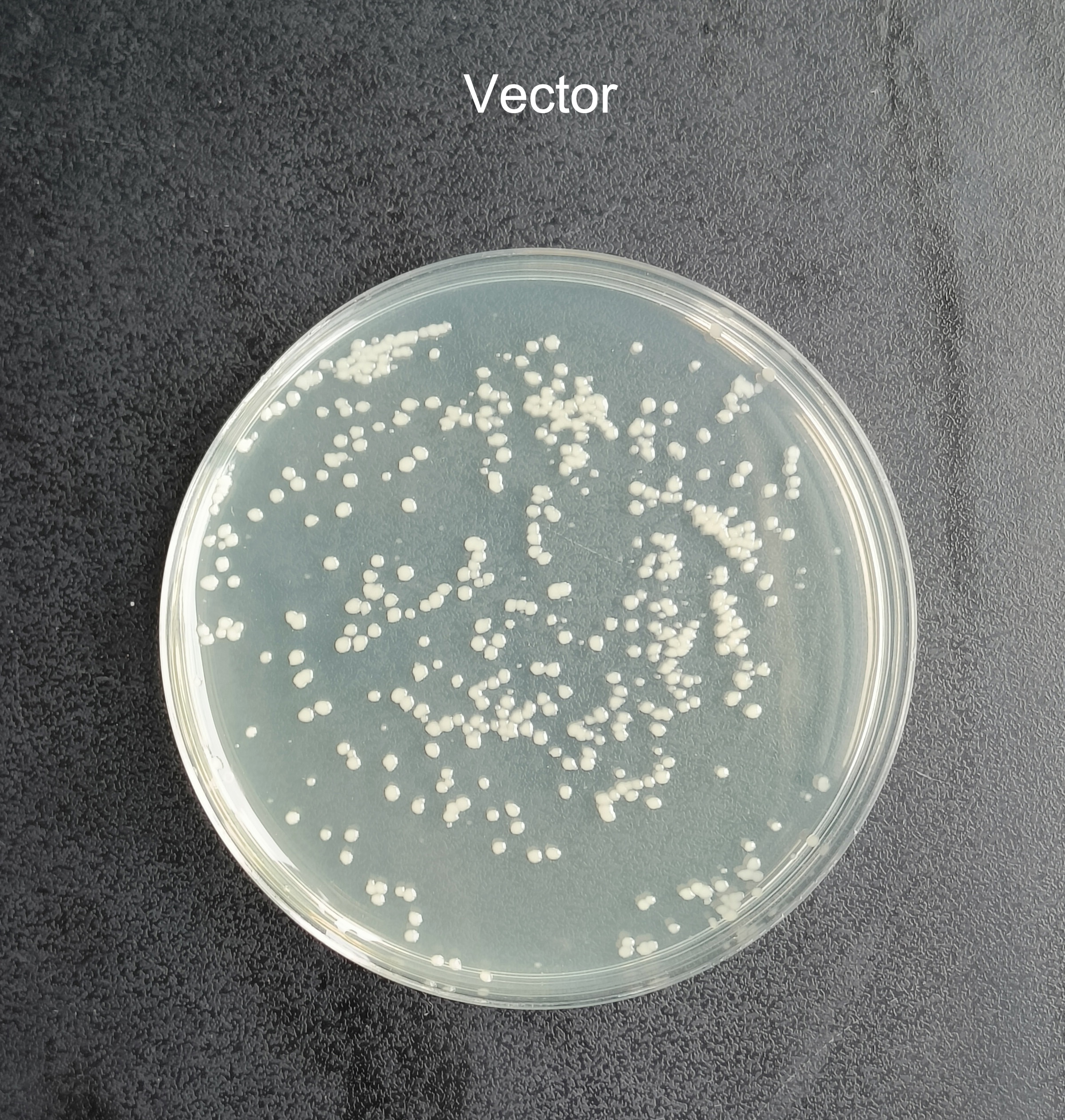

Supplement: Supplementary file 6 — Source data Fig. 4 [file 44319_2025_379_MOESM6_ESM.zip › Figure 4/4K/4K-vector.jpg]

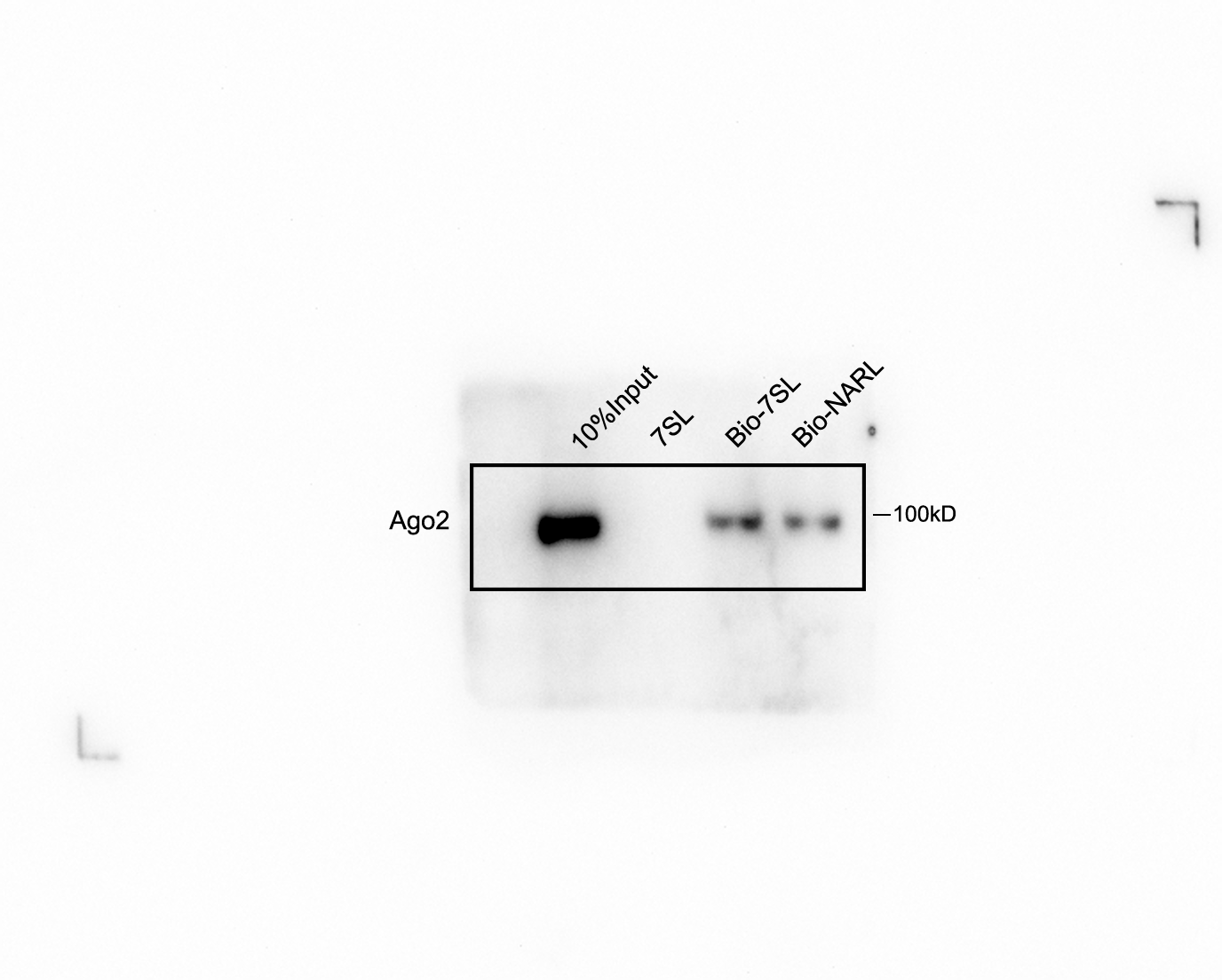

Supplement: Supplementary file 7 — Source data Fig. 5 [file 44319_2025_379_MOESM7_ESM.zip › Figure 5/5A/5A.tif]

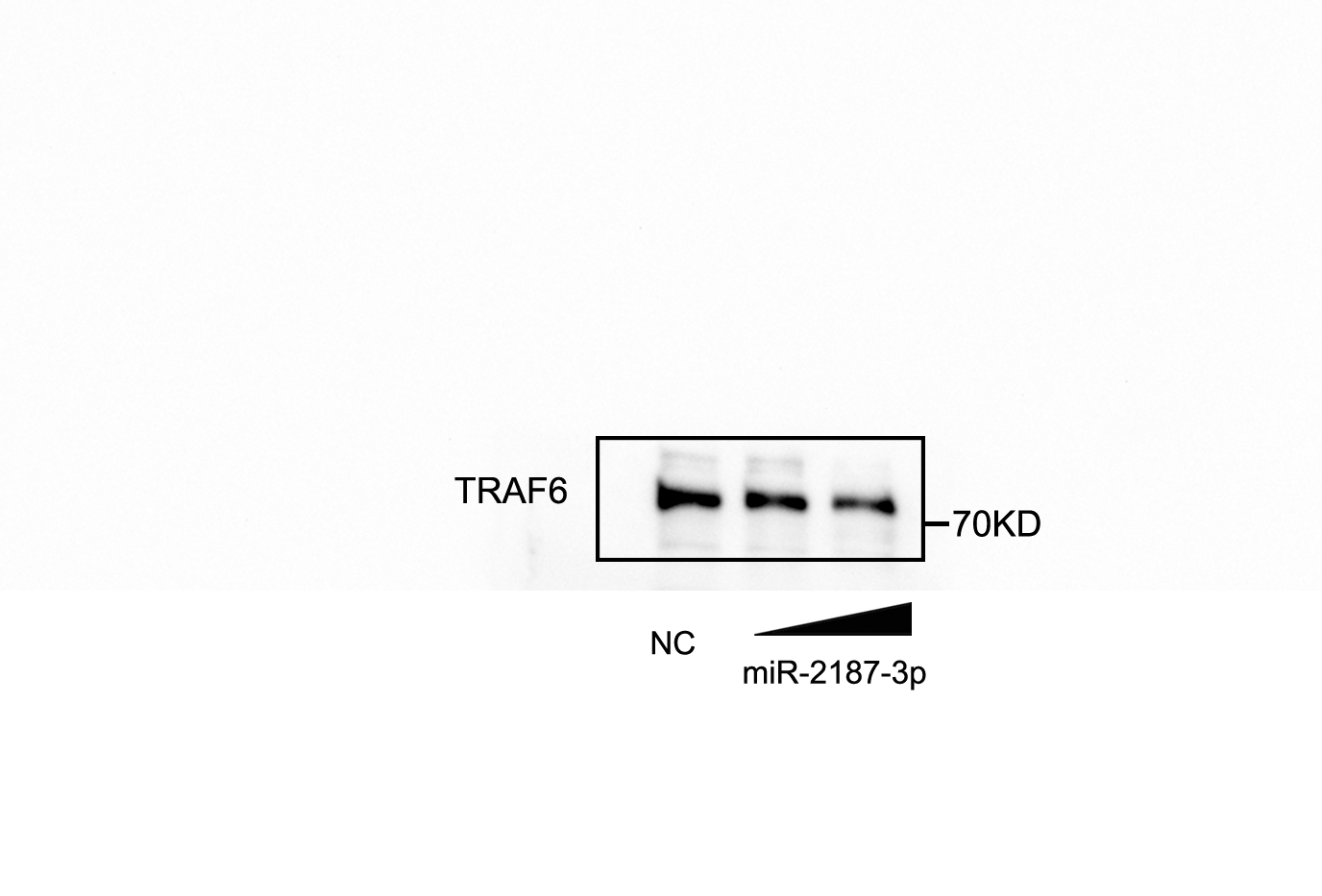

Supplement: Supplementary file 7 — Source data Fig. 5 [file 44319_2025_379_MOESM7_ESM.zip › Figure 5/5K/5K-left-TRAF6.tif]

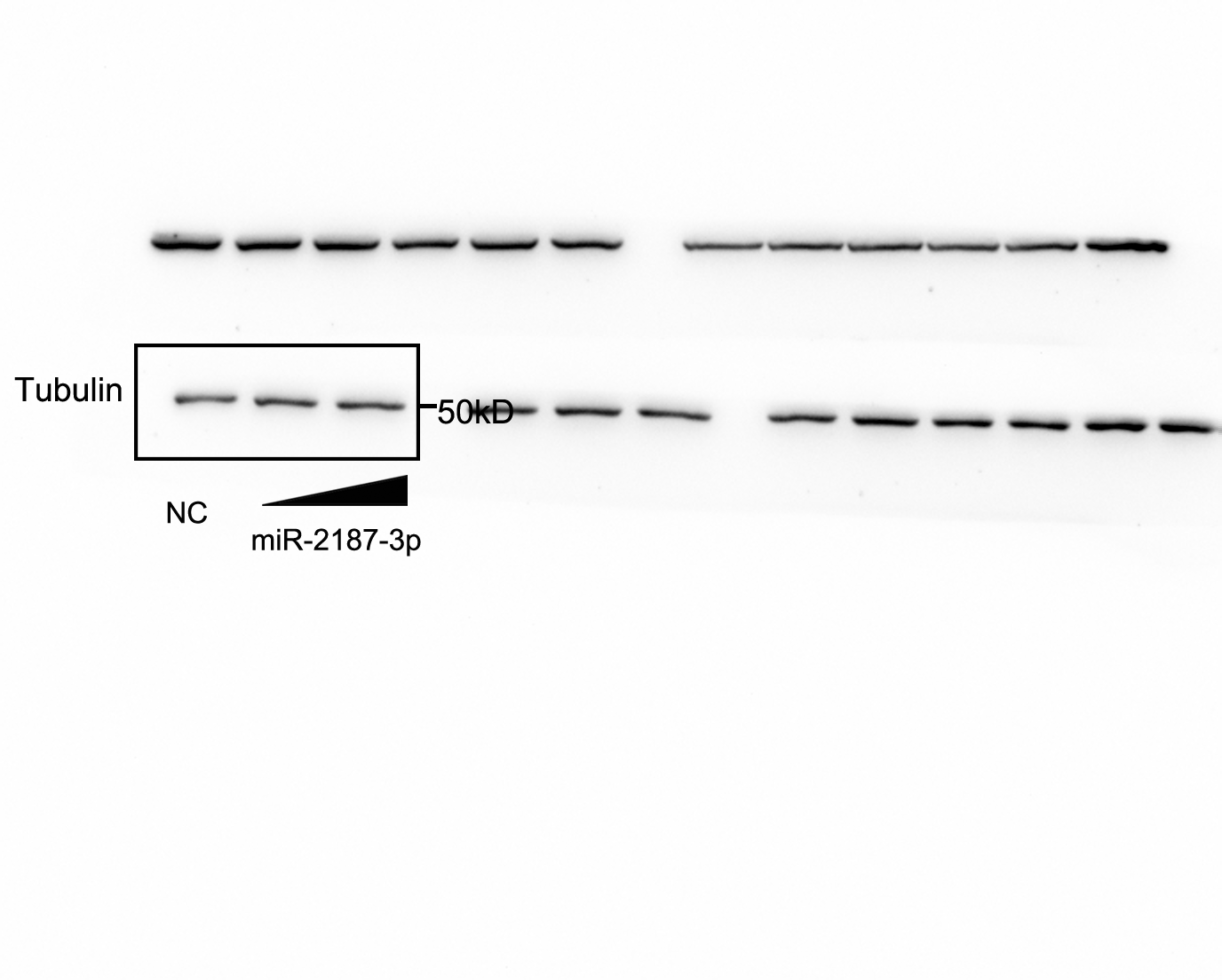

Supplement: Supplementary file 7 — Source data Fig. 5 [file 44319_2025_379_MOESM7_ESM.zip › Figure 5/5K/5K-left-Tubulin.tif]

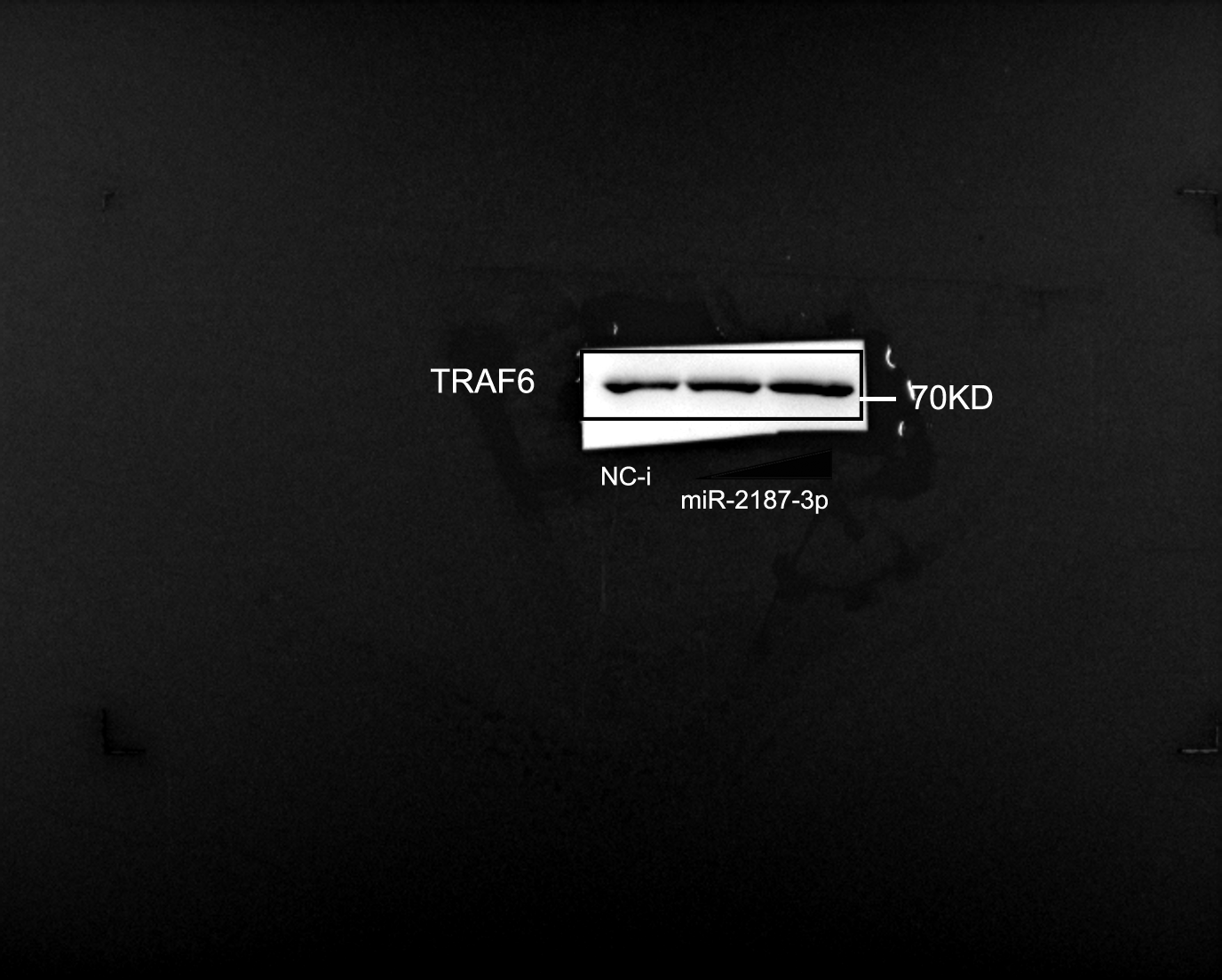

Supplement: Supplementary file 7 — Source data Fig. 5 [file 44319_2025_379_MOESM7_ESM.zip › Figure 5/5K/5K-right-TRAF6.tif]

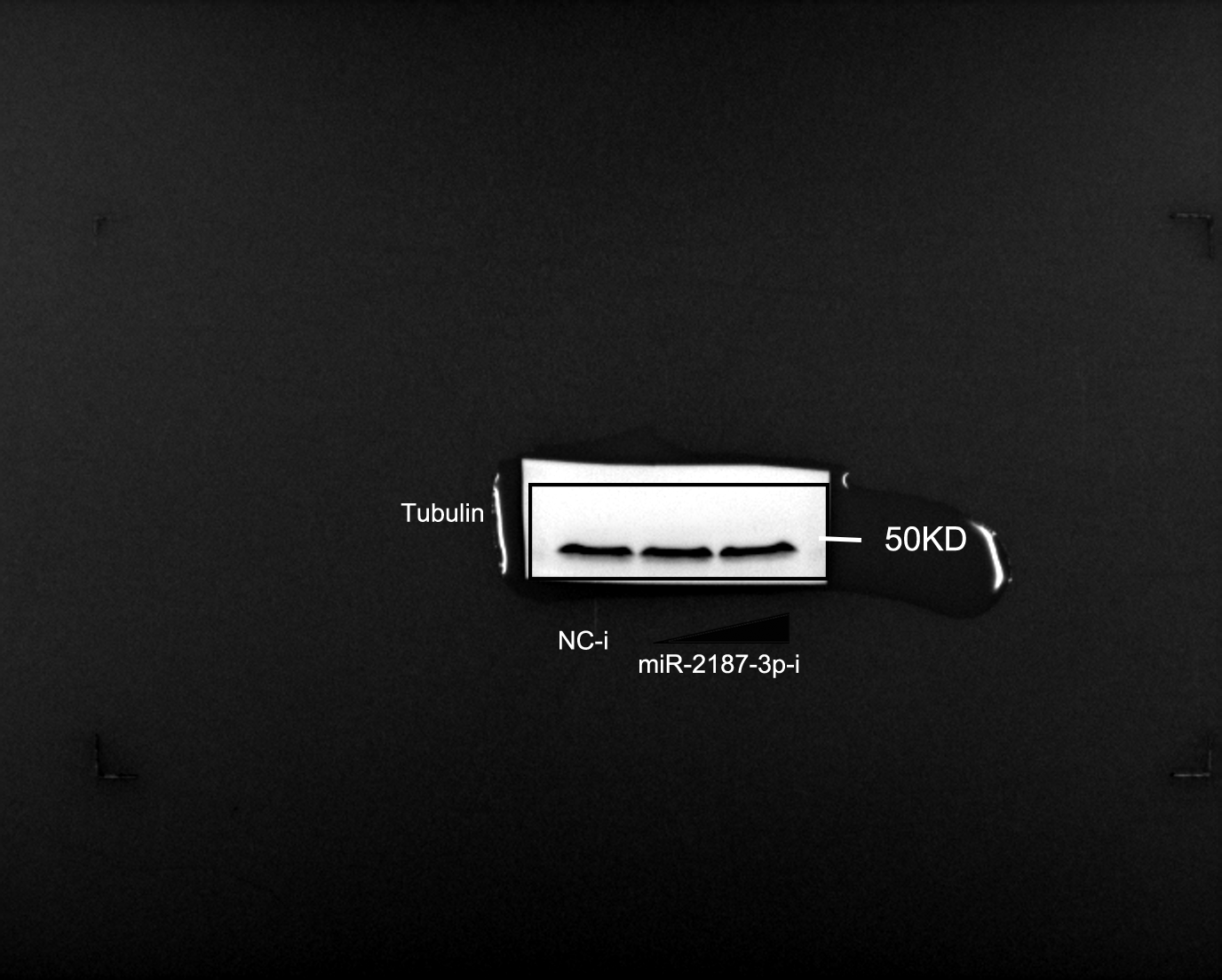

Supplement: Supplementary file 7 — Source data Fig. 5 [file 44319_2025_379_MOESM7_ESM.zip › Figure 5/5K/5K-right-Tublin.tif]

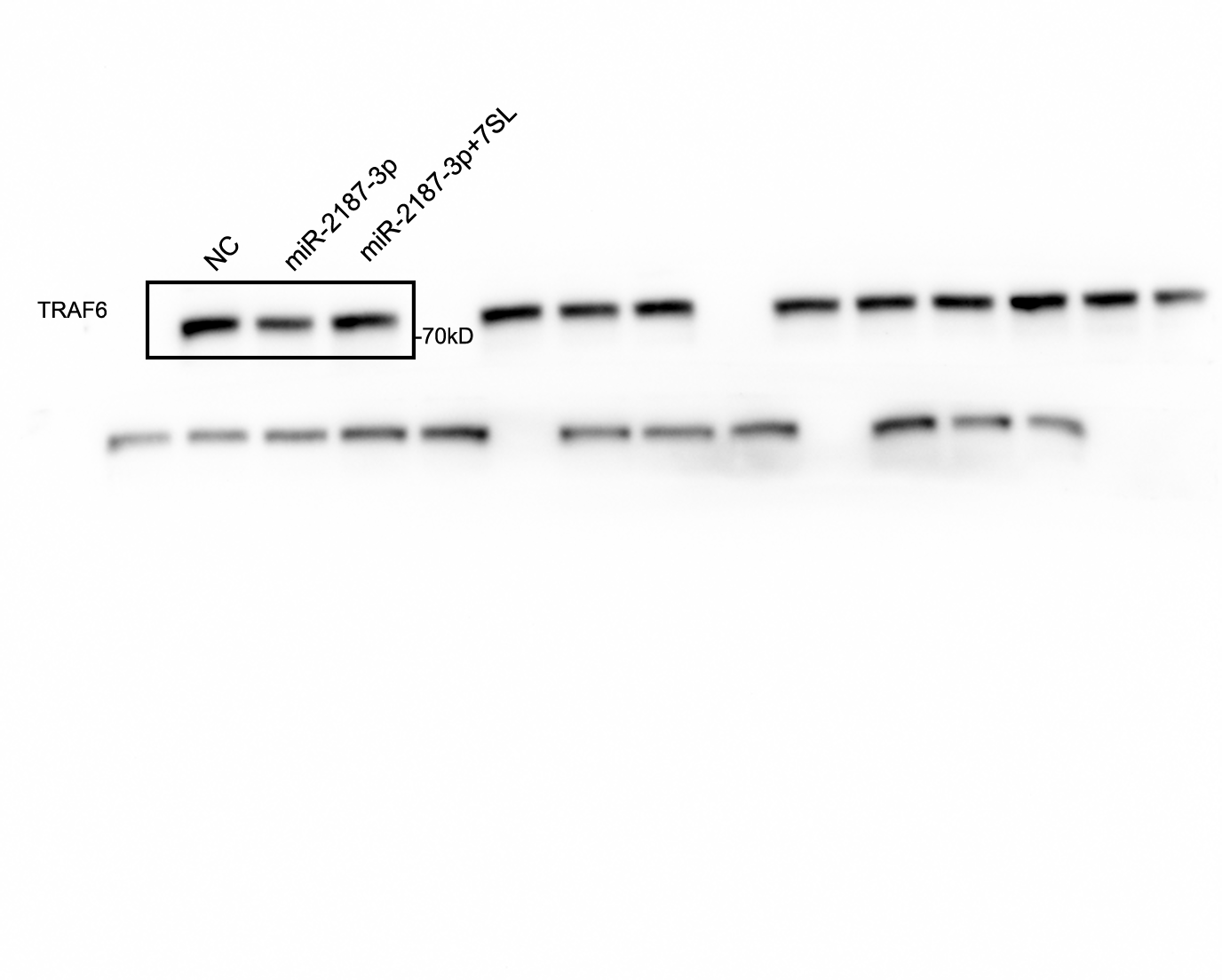

Supplement: Supplementary file 7 — Source data Fig. 5 [file 44319_2025_379_MOESM7_ESM.zip › Figure 5/5L/5L-TRAF6.tif]

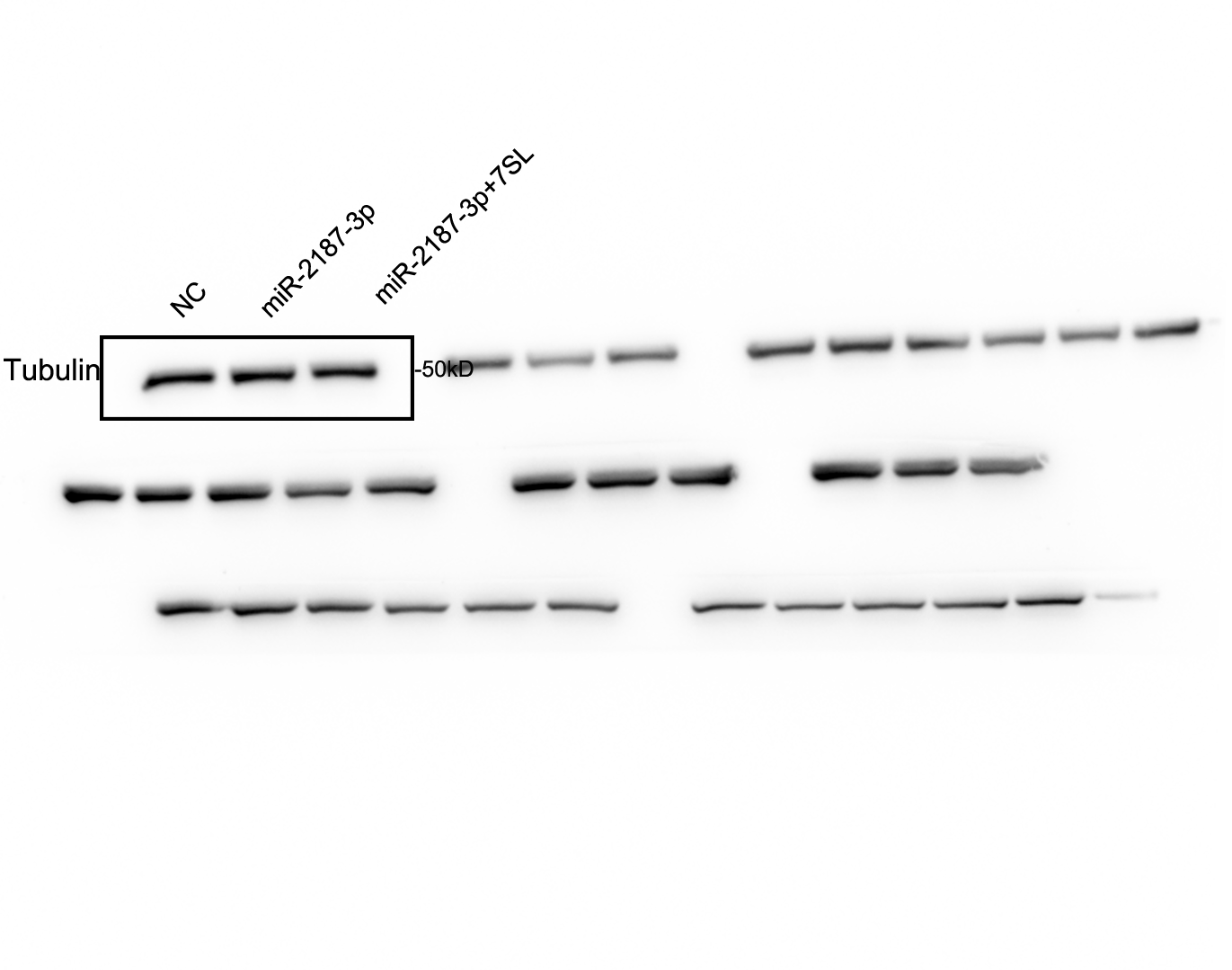

Supplement: Supplementary file 7 — Source data Fig. 5 [file 44319_2025_379_MOESM7_ESM.zip › Figure 5/5L/5L-Tubulin.tif]

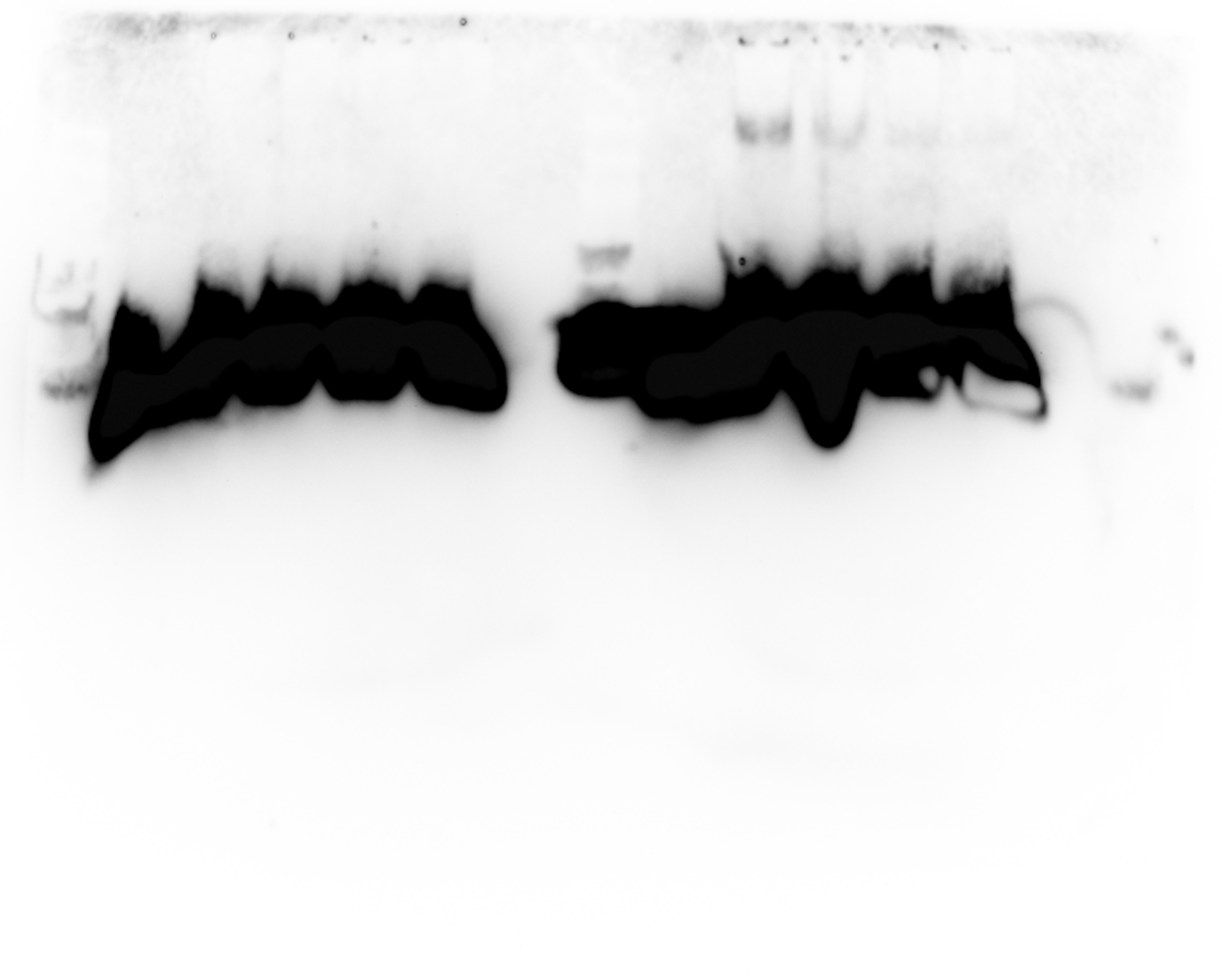

Supplement: Supplementary file 8 — Figure EV1 Source Data [file 44319_2025_379_MOESM8_ESM.zip › Figure EV1/120.Tif]

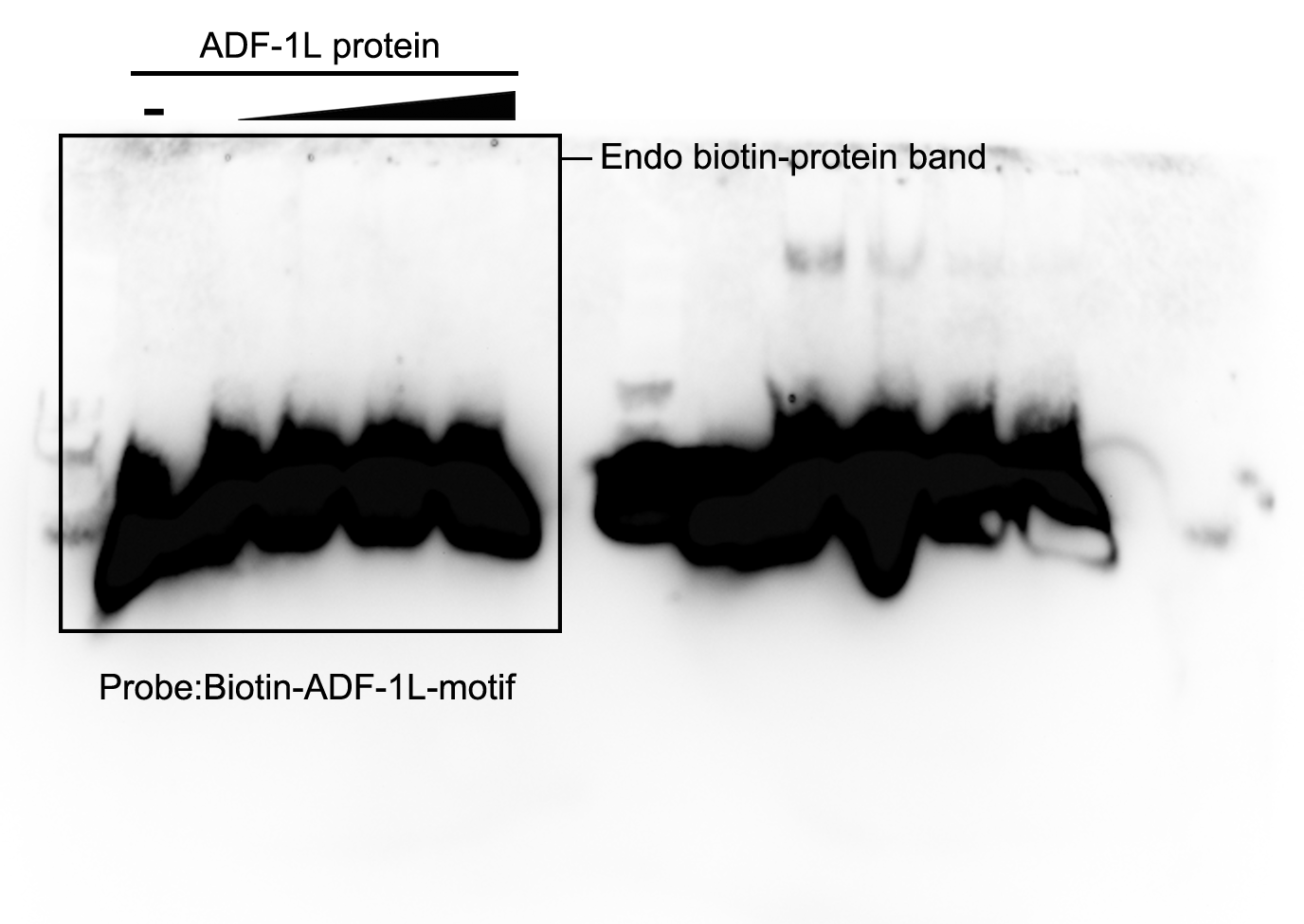

Supplement: Supplementary file 8 — Figure EV1 Source Data [file 44319_2025_379_MOESM8_ESM.zip › Figure EV1/EV1C.tif]
