# Supplementary material for: PIF/harbinger transposon-derived protein promotes 7SL expression to enhance pathogen resistance
Source: EMBO Rep. 2025 Jan 30;26(5):1196–211. doi: 10.1038/s44319-025-00379-8 (PMC11893794; doi:10.1038/s44319-025-00379-8)
Supplement: Supplementary file 9 — Expanded View Figures [file 44319_2025_379_MOESM9_ESM.pdf]

Expanded View Figures

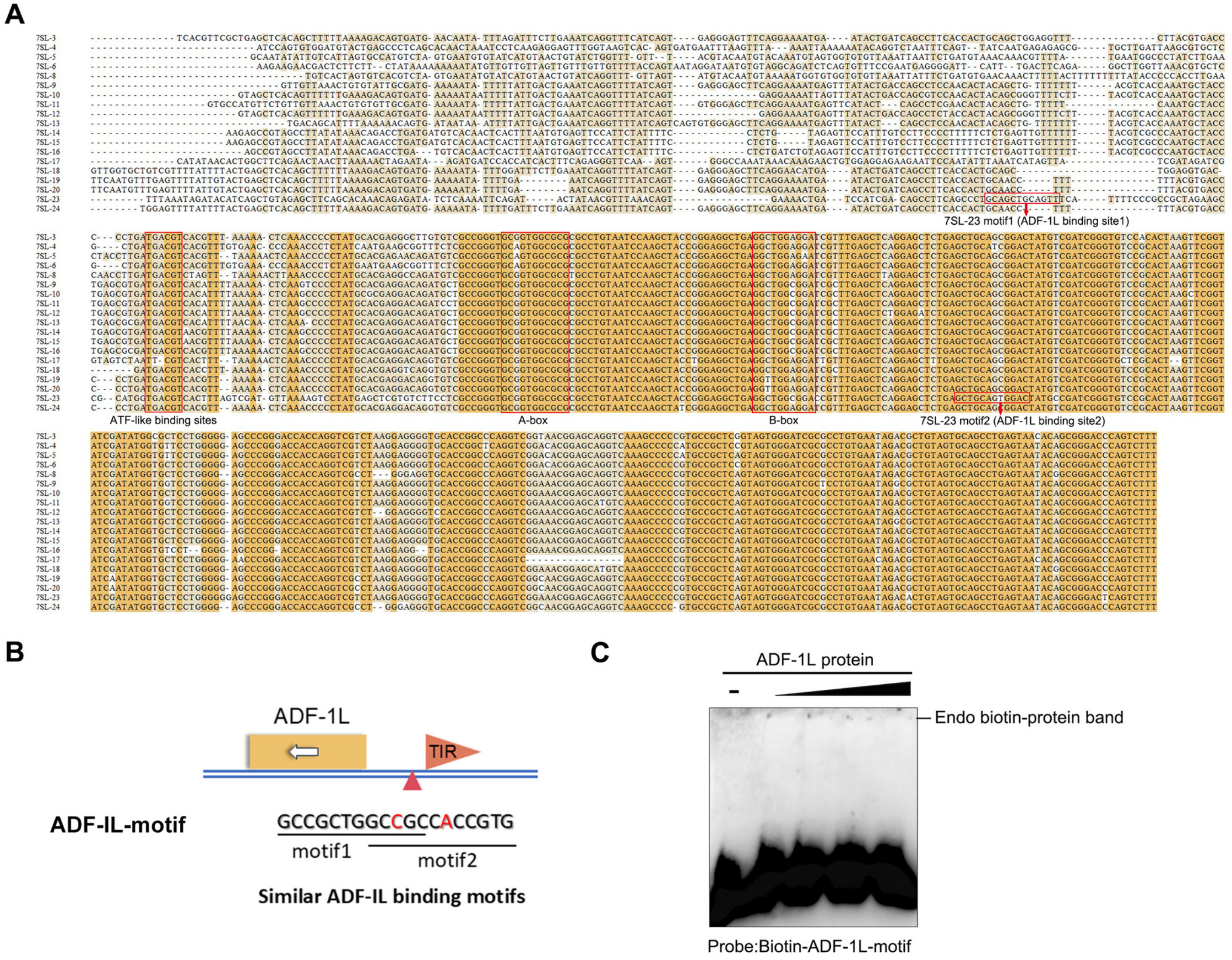

Figure EV1. Presentation of ADF-IL cis-binding sites.

(**A**) The sequence alignment of 19 7SL gene with A box and B box (internal sequence + 200 bp external sequence), and the specific motif bound by ADF-IL has been labeled in 7SL-23. (**B**) A sequence of ADF-IL promoter containing two similar ADF-1L binding motifs. (**C**) EMSA assay performed with increasing concentrations of ADF-1L protein, and biotin-labeled probes (similar ADF-1L-motif). Source data are available online for this figure.

 © The Author(s)
